# Pairing of Homologous Regions in the Mouse Genome Is Associated with Transcription but Not Imprinting Status

**DOI:** 10.1371/journal.pone.0038983

**Published:** 2012-07-03

**Authors:** Christel Krueger, Michelle R. King, Felix Krueger, Miguel R. Branco, Cameron S. Osborne, Kathy K. Niakan, Michael J. Higgins, Wolf Reik

**Affiliations:** 1 Epigenetics Programme, The Babraham Institute, Cambridge, United Kingdom; 2 Bioinformatics Group, The Babraham Institute, Cambridge, United Kingdom; 3 Genome Function Group, MRC Clinical Sciences Centre, Imperial College School of Medicine, Hammersmith Hospital Campus, London, United Kingdom; 4 Nuclear Dynamics Programme, The Babraham Institute, Cambridge, United Kingdom; 5 Centre for Trophoblast Research, University of Cambridge, Cambridge, United Kingdom; 6 Anne McLaren Laboratory for Regenerative Medicine, Department of Physiology, Development and Neuroscience, University of Cambridge, Cambridge, United Kingdom; 7 Department of Molecular and Cellular Biology, Roswell Park Cancer Institute, Buffalo, New York, United States of America; Institut Curie, France

## Abstract

Although somatic homologous pairing is common in *Drosophila* it is not generally observed in mammalian cells. However, a number of regions have recently been shown to come into close proximity with their homologous allele, and it has been proposed that pairing might be involved in the establishment or maintenance of monoallelic expression. Here, we investigate the pairing properties of various imprinted and non-imprinted regions in mouse tissues and ES cells. We find by allele-specific 4C-Seq and DNA FISH that the *Kcnq1* imprinted region displays frequent pairing but that this is not dependent on monoallelic expression. We demonstrate that pairing involves larger chromosomal regions and that the two chromosome territories come close together. Frequent pairing is not associated with imprinted status or DNA repair, but is influenced by chromosomal location and transcription. We propose that homologous pairing is not exclusive to specialised regions or specific functional events, and speculate that it provides the cell with the opportunity of *trans*-allelic effects on gene regulation.

## Introduction

Tight spatial and temporal regulation of gene expression requires several interleaved layers of control. Apart from protein factor binding to *cis* regulatory regions, modifications of DNA and chromatin, position of the gene in nuclear space, and an intricate network of chromosome associations in *trans* determine the expression state of a particular gene. Often, co-regulated genes are found in the same transcription factory, bringing together various regions from different chromosomes [1]. This is, however, not limited to heterologous regions. In fact, pairing of homologous chromosomes has long been known in *Drosophila*. It underlies the phenomenon of transvection which refers to changes in gene activity through interaction of regulatory elements of one allele with its homologue [2]. Although somatic pairing of whole chromosomes is not generally observed in mammalian cells, homologous pairing is documented in a number of studies. The most prominent example refers to the interaction of the two X chromosomes at the onset of X inactivation which is thought to break symmetry and destine one partner for silencing [3]. Interestingly, other monoallelically expressed regions have also been shown to pair: immunoglobulin loci interact during recombination which is thought to contribute to the process of allelic exclusion [4]. Moreover, homologous pairing was demonstrated for the Prader-Willi/Angelman imprinted region in human lymphocytes and brain [5], [6]. It has been proposed that somatic pairing is a general feature of regions for which one allele is silenced which might be involved in the establishment or maintenance of monoallelic expression [4], [7], [8].

However, not all examples of homologous pairing in somatic cells involve monoallelically expressed regions. Renal oncocytoma cells show pairing of the q arms of chromosome 19 as a chromosomal abnormality associated with misregulation of gene expression [9]. Also, pairing of subtelomeric regions was observed in a human fibroblast cell line and primary lymphoblasts which might play a role in cytogenetically cryptic deletions and translocations involving chromosome ends [10]. The likelihood of homologous pairing is also affected by the radial position of the chromosome which is in turn dependent of chromosome size, gene density, transcriptional activity and presence of nucleolus organiser regions (NORs) [11]–[14]. As an alternative explanation, it can therefore be argued that pairing events are side-effects of large scale chromosomal features. It is currently unclear what drives homologous associations: They may be the consequence of specific interactions between defined genomic elements, or alternatively may be caused by the properties of large chromosomal regions. In this study, we have investigated in detail the pairing properties of various imprinted and non-imprinted genomic regions, and explored the possibility that pairing occurs for a specific purpose outside specialised settings such as X-inactivation or allelic exclusion. We find pairing frequency to be dependent on chromosomal position and transcriptional activity, rather than on mono- or biallelic expression of genes. We propose that homologous pairing is an infrequent but widespread phenomenon which may in certain situations open up the opportunity of two alleles communicating in *trans* to regulate gene expression.

## Results

### Homologous Pairing is Observed at the *Kcnq1* Imprinting Region

**Figure 1:**
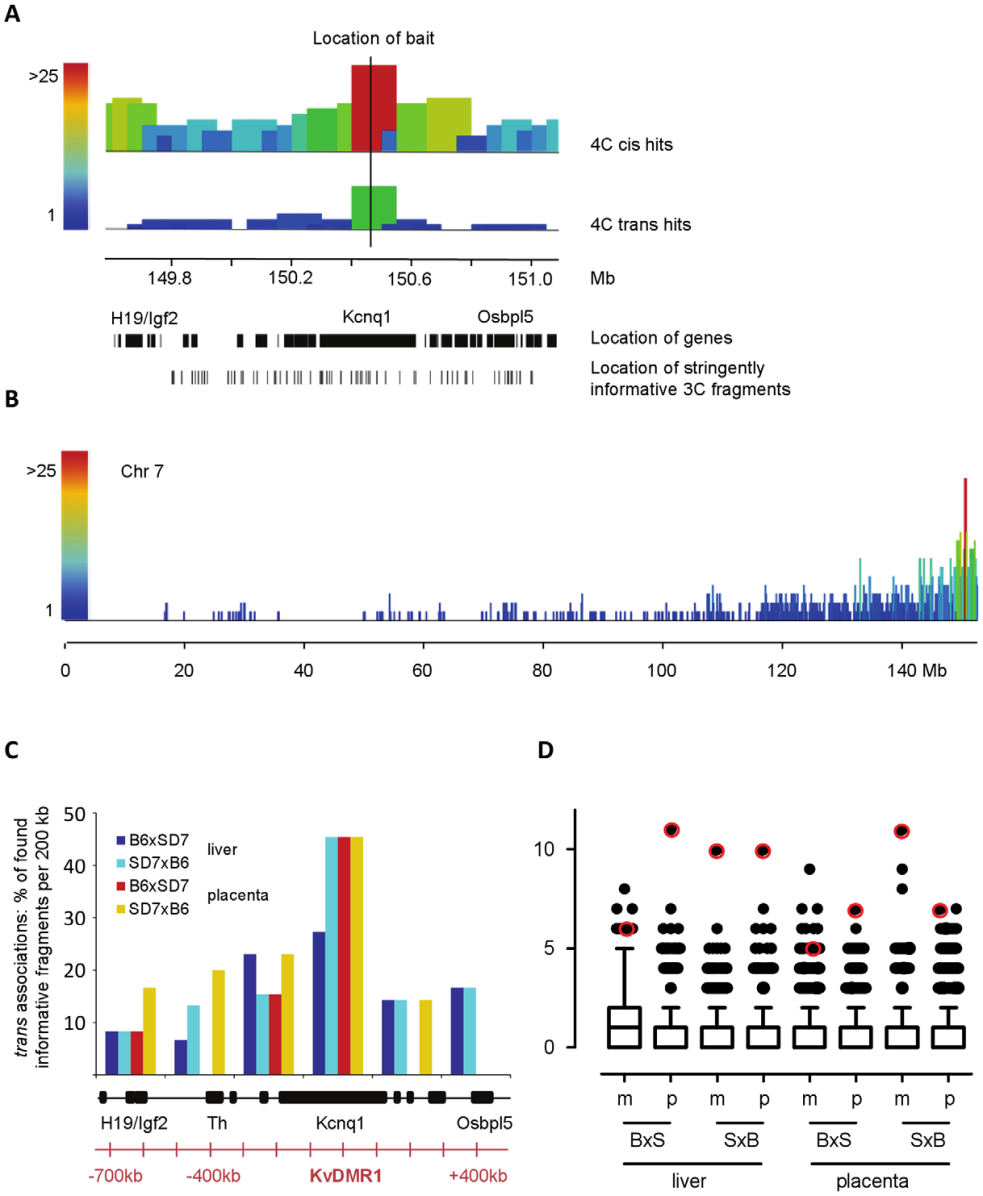
4C-Seq reveals *trans*-allelic associations. **A**) Example of 4C-Seq association profile surrounding the KvDMR bait in the middle of the *Kcnq1* gene (window size 1.5 Mb, sample B6xSD7, E13.5 liver). The first row shows the quantification of all non-duplicated 4C-Seq reads per 100 kb window for the maternal bait in *cis*. 4C-Seq reads can only occur at certain positions and each position was counted only once (see File S2 for details). Colour and height of the bar reflect how many positions were found per 100 kb window (31 for the window that includes the bait). The second row shows associations of the maternal bait (B6) with the *trans* (paternal, SD7) allele. All reads identified as SD7 specific by ASAP were quantified per 100 kb window (see File S2 for details), again counting every position only once. A scale bar indicates the location of the region on chromosome 7. Black bars below represent the location of genes with some labelled for orientation. The positions of 3C fragments classified as ‘stringently informative’ are depicted at the bottom (see File S2 for details). The vast majority of associations occurs in *cis*. *Trans*-allelic associations occur most frequently with the homologous region. **B**) Overview over *cis* associations on chromosome 7 (sample B6xSD7, E13.5 liver, maternal bait, 100 kb windows, read positions counted only once, zoomed out from 1A). Height and colour of bars reflect how many read positions were found per 100 kb window. The bait is located near the telomeric end of chromosome 7. 4C-Seq reads are most frequent around the bait and drop away with increasing genomic distance. **C**) Summary of *trans*-allelic associations for E13.5 liver and placenta for different parental crosses. For each 200 kb window a certain number of 3C fragments are stringently informative for their allelic origin (see File S2 for details). If a stringently informative fragment was found in a particular data set and identified as a *trans*-association with either the maternal or the paternal bait, it was counted as a hit. *Trans*-allelic associations per window are displayed as hits divided by the number of stringently informative fragments. If no bar is displayed, no *trans*-allelic associations were identified for this window. Below, locations of genes and distances from the KvDMR bait are indicated. For all samples, *trans*-allelic associations peak around the KvDMR region on the other allele. **D**) Comparison of homologous *trans*-allelic 4C-Seq associations with non-homologous *trans* associations in the genome. The genome was split into 1.4 Mb windows (matching the size of the chromosome 7 region carrying allelic information) and unique *trans* associations of the KvDMR bait with regions within these windows were counted. Data are shown as Tukey box-whisker plots. Data points marked by a red circle represent *trans*-allelic hits to the corresponding region on the other chromosome. m: maternal bait, p: paternal bait, BxS: cross B6xSD7, SxB: cross SD7xB6. Most regions do not associate with the KvDMR bait, however, some regions are over represented (outliers). *Trans*-allelic associations occur with a similar frequency to other non-homologous *trans* associations.

The *Kcnq1* cluster is a large imprinted region located on distal mouse chromosome 7. Imprinted protein-coding genes of this cluster are expressed from the maternal allele, while a long non-coding RNA expressed from the paternal allele covers the locus to create a repressive compartment [15]–[17]. The maternal allele carries a germline methylation mark in the locus control region (KvDMR1), and monoallelic expression of the non-coding RNA is set up by the two cell stage [18]. Imprinted expression is maintained throughout development, but interestingly extra-embryonic tissues display a larger monoallelic region and different chromatin features than the embryo proper [19], [20]. Since homologous pairing was previously proposed to be linked to monoallelically expressed regions [4], [5], we probed the *Kcnq1* imprinted region for interactions with its homologous allele. We adapted the linear 4C technique [21] to use with high throughput sequencing (abbreviated 4C-Seq, see File S1 for details). Using a cross between C57BL/6J and a congenic strain carrying the distal part of chromosome 7 from *Mus spretus* (SD7) results in a subset of 3C restriction fragments being informative for their allelic origin (Fig. 1A, for details see Materials and Methods). One of these fragments is located in the central imprinting control region (KvDMR) and was chosen as bait, rendering the 4C interaction profiles allele-specific. As anticipated, the vast majority of interactions occurs on the *cis* allele but strikingly, we also found a number of chimeric 4C products consisting of a maternal bait and a paternal prey, or vice versa (Fig. 1A). This suggests that for both tissues analysed (E13.5 foetal liver and placenta to represent embryonic and extra-embryonic lineages) the two homologous alleles were in close proximity at the time of cross-linking. The highest frequency of *trans* allelic interactions is found with the corresponding region on the other allele which is indicative of homologous pairing (Fig. 1B). Interestingly, the frequency of homologous interactions is in the upper range of all *trans* interactions genomewide (Fig. 1C). This demonstrates that although rare compared to *cis* interactions, homologous associations are just as prevalent as other heterologous *trans* interactions which can be functionally relevant, such as preferential associations between co-regulated genes in transcription factories [21].

**Figure 2:**
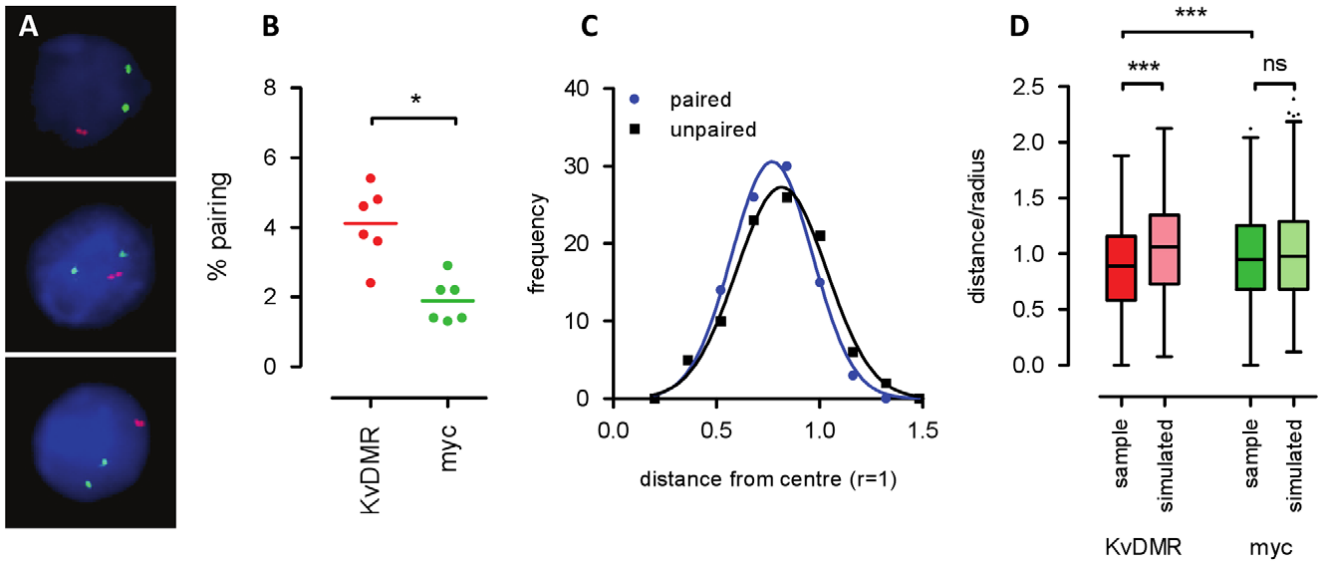
DNA FISH confirms high pairing frequency for the KvDMR region. **A**) Examples of paired DNA FISH spots in E13.5 foetal liver. Red: probe covering the KvDMR region, green: probe covering the region around the myc gene, blue: DAPI counterstain. **B**) Pairing frequency of the KvDMR and myc regions. Each dot represents one biological sample, and for each sample 300 nuclei were counted in four technical replicates. Differences were assessed by unpaired t-test, ns: not significant, *: p<0.05, ***: p<0.001. KvDMR signals show higher pairing frequency than myc signals. **C**) Frequency distributions of radial distances of unpaired (black) and paired (blue) KvDMR DNA FISH signals in E13.5 liver (n = 93). Radial distances >1 can occur if the nucleus is not a perfect sphere. Radial distances are not different between paired and unpaired KvDMR FISH signals (t-test, p = 0.0668) indicating that pairing events can happen at all nuclear locations KvDMR alleles normally occupy. **D**) Distances between homologous alleles in E13.5 liver represented as Tukey box-whisker plots (n = 600). Interallelic distances were normalised to the radius of the nucleus (distance/radius). Differences were assessed by one-way ANOVA with Bonferroni’s multiple comparison post test. Simulated: a group of spots displaying the same radial distributions as KvDMR and myc FISH signals, respectively, were placed into a sphere at random and their interallelic distances determined (see Fig. S1A, B and Methods). While interallelic distances between myc signals show a distribution expected from their radial positions (no difference between sample and simulated, p>0.05), KvDMR signals are significantly closer together than expected (difference between sample and simulated, p<0.001). Also, KvDMR signals are generally closer together than myc signals. The same data is represented as a histogram in Fig. S1C to illustrate the presence of a subpopulation of KvDMR signals that display very short interallelic distances.

We then set out to validate our 4C-Seq results by 3D DNA FISH. We used an automated image acquisition system to be able to capture rare events and carefully defined conservative scoring criteria to minimise confounding effects (Fig. 2A, see Materials and Methods for details). For a probe covering the centre region of the *Kcnq1* imprinted region (KvDMR) we find pairing events in around 4% of foetal liver nuclei, twice as often as for a control probe covering a region around the *myc* gene (myc, Fig. 2B). To evaluate if pairing events happened at particular regions in the nucleus, we determined radial positions of paired and unpaired KvDMR alleles. No significant difference was found indicating that pairing events can happen at all positions KvDMR alleles usually occupy (Fig. 2C). We then compared 3D inter-allelic distances between two homologous alleles. Strikingly, KvDMR alleles were significantly closer together than myc alleles (Fig. 2D). To analyse if this was due to differences in radial distributions for KvDMR and myc alleles, we simulated FISH signals reflecting the radial distributions of KvDMR and myc alleles, respectively, but which are otherwise random (see Fig. S1 and Materials and Methods for details). While interallelic distances between observed myc alleles were not different from distances of simulated myc alleles, observed KvDMR alleles showed significantly reduced interallelic distances compared to their simulated counterparts. This confirms that KvDMR alleles are generally closer together than myc alleles, and that this is not due to their different radial distributions in the nuclear space. The low frequency of *trans* allelic 4C products and paired DNA FISH spots suggests that homologous pairing is a transient event, and that overall shorter distances between KvDMR alleles are observed because the regions are on their way in or out of an interaction. Alternatively, homologous pairing might be an infrequent but relatively stable spatial arrangement in a subset of cells potentially causing variegated expression [22]. Remarkably, very similar data were obtained from ES cells (Fig. S2) which reflect well the imprinting properties of the embryonic lineage at the *Kcnq1* locus but are known to have a very different genome organisation from differentiated cells. Presence of *trans* allelic interactions in diverse tissues like foetal liver and placenta, as well as undifferentiated ES cells demonstrates that homologous pairing is not restricted to specific tissues or cell types.

### Pairing can Involve Extended Chromosomal Regions and Brings Homologues Close Together

**Figure 3:**
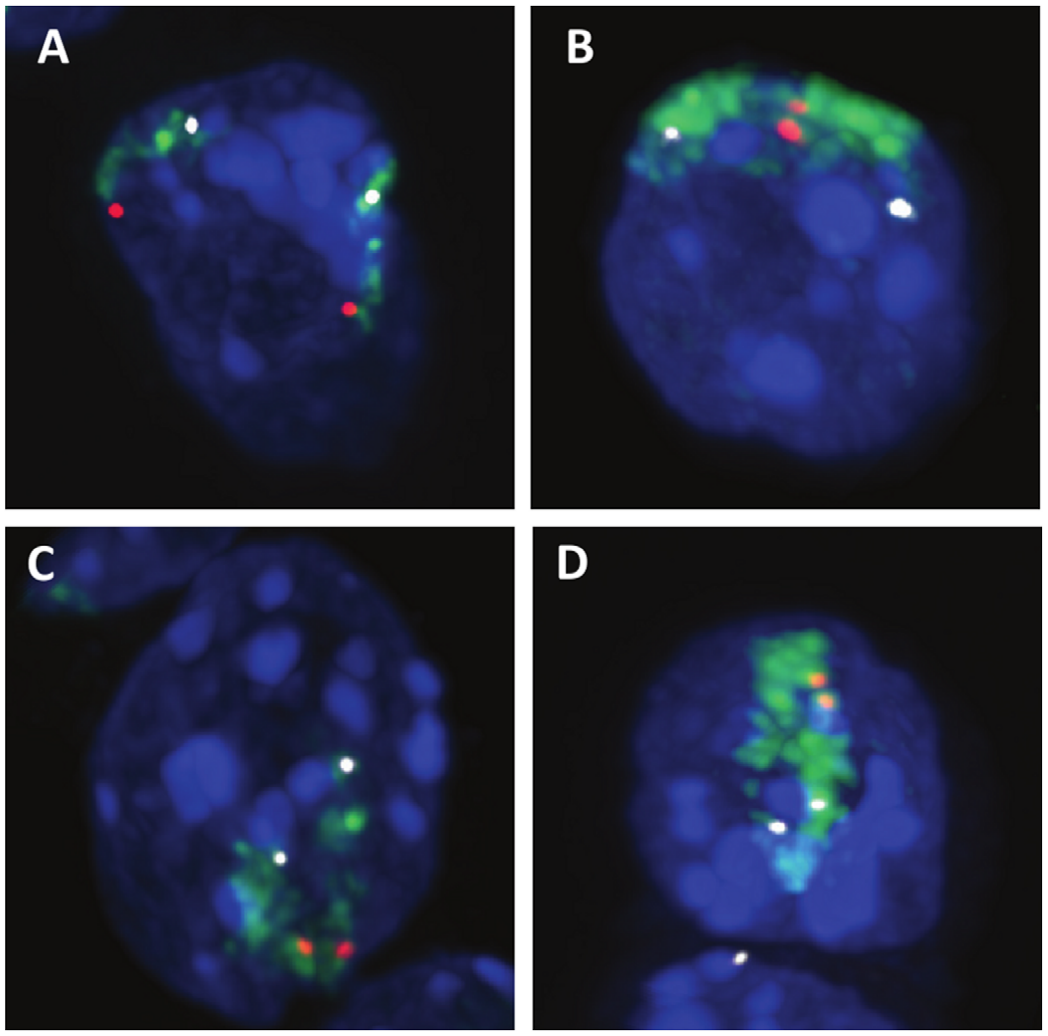
Regional pairing brings chromosome territories close together. 3D representation of stacks of confocal images from ES cells. Green: chromosome 7 painting, red: DNA FISH with a probe covering KvDMR near the distal end of chromosome 7, white: DNA FISH with a probe covering *Kcnn4* 25 Mb away from the proximal end of chromosome 7. FISH signals are mostly located in or close to the edge of their chromosome territory. Most nuclei display two separate chromosome 7 territories (**A**). When KvDMR signals are close together, the territory arrangements can be ‘touching’ (**B**) with the proximal ends pointing away from each other, ‘Y-shaped’ with a larger region aligning (**C**), or ‘aligned’ with most of the chromosome being parallel (**D**). These arrangements occur with very similar frequencies (n = 9, 8, 9, respectively).

To find out if pairing events happened for isolated loci or if larger regions of the chromosomes would come into close proximity, we used whole chromosome painting together with probes marking the KvDMR region and a region near the centromeric end of chromosome 7. Most nuclei showed two separate chromosome 7 domains which tended to be positioned away from each other (Fig. 3A, Movie S1). When KvDMR signals were paired, they were observed within or close to the edge of their chromosome territory with the two chromosome 7 domains coming together. We found three sub-classes of KvDMR pairing with equal frequency (Fig. 3B–D): i) ‘Touching’: the chromosome territories overlap only in the region surrounding KvDMR. The centromeric ends point away from each other. ii) ‘Y-shaped’: the chromosome territories overlap in the region surrounding KvDMR and the overlap extends further along the chromosome. iii) ‘Aligned’: the two homologous chromosomes are more or less aligned along the entire length of the chromosome. In conclusion, pairing does not involve isolated regions which form large loops to contact their homologue, but rather affects larger chromosomal regions.

**Figure 4:**
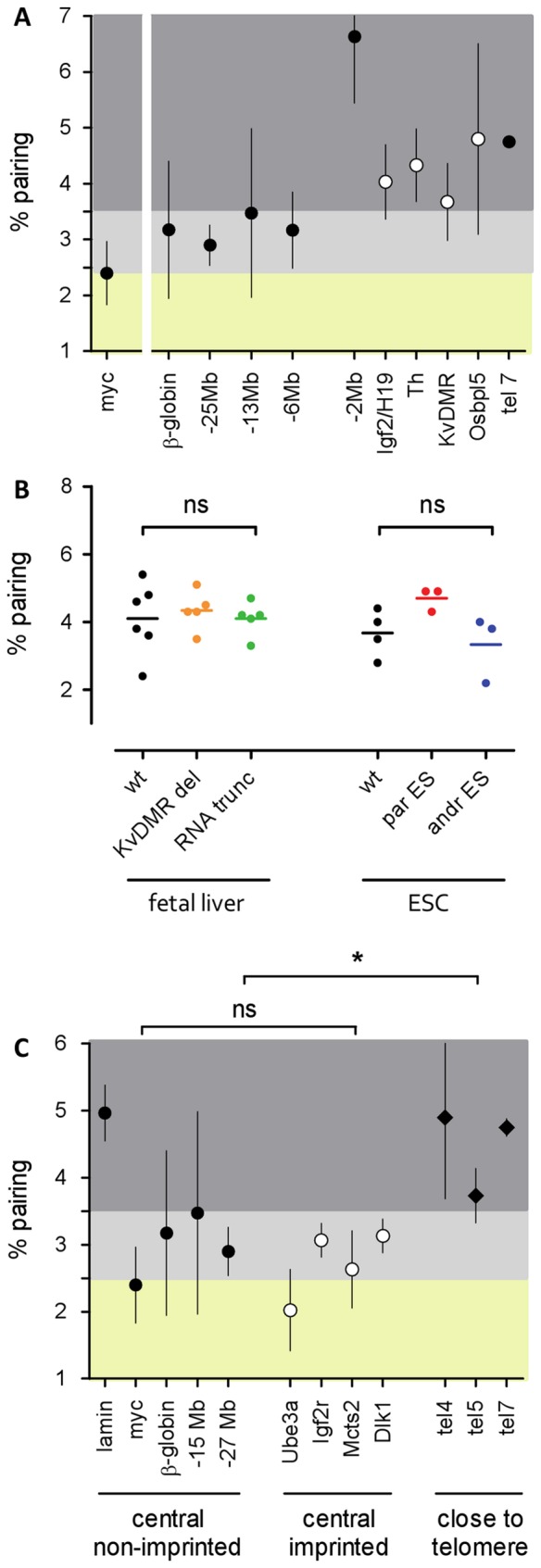
Pairing is not limited to monoallelically expressed regions. **A**) Extent of paired region on chromosome 7 in ES cells. Each dot represents the mean of three to four samples taken from different passages with the pairing frequency determined for each sample in four technical replicates of 300 nuclei. Whiskers represent standard deviation. Probes from the imprinted region on distal chromosome 7 are shown as open circles, probes from non-imprinted regions are shown as filled circles. Background shading indicates high pairing frequency (dark grey, above 3.5%), medium (light grey, 2.5–3.5%) and low pairing frequency (yellow, below 2.5%). The myc probe (chromosome 15) is displayed for comparison. Frequent pairing is observed for the distal end of chromosome 7 but not limited to the imprinted region. **B**) Pairing in imprinting deficient mutants. Each dot represents one biological sample with the pairing frequency determined in four technical replicates of 300 nuclei. The mean is represented by a line. Differences were assessed by unpaired t-test, ns: not significant. Wt: wildtype, KvDMR del: paternal deletion of KvDMR abolishing regional silencing and removing functionally important CTCF binding sites, RNA trunc: paternal truncation of the non-coding RNA Kcnq1ot1 responsible for monoallelic silencing, par ES: parthenogenetic ES cells harbouring two maternal genomes, andr ES: androgenetic ES cells harbouring two paternal genomes. Neither the monoallelic expression state nor the presence of a biparental genome is a prerequisite for frequent pairing in the region. **C**) Pairing of other imprinted and telomeric regions in ES cells. For a description of data points and background see A. Differences were assessed by unpaired t-test, ns: not significant, *: p<0.05. Imprinted regions are represented by open circles, regions close to the telomere by filled diamonds. Probes covering genes in various imprinted regions do not display high pairing frequency in contrast to probes located near telomeres.

In order to determine the extent of the paired region around KvDMR, we placed several FISH probes within the *Kcnq1* imprinted region, and further away along chromosome 7 (Fig. 4A). All probes within the imprinted region showed high pairing frequencies, but homologous associations did not stop at the boundaries: probes on either side of the imprinted locus presented with a high number of paired FISH spots as well. However, probes from 6 to 39 Mb away from the imprinted locus showed reduced pairing frequency. In accordance with the chromosome painting data this suggests that homologous pairing occurs over larger chromosomal regions but does not uniformly affect whole chromosomes.

### Pairing is not Linked with Monoallelic Expression

We next aimed to determine if the mono-allelic expression state of the *Kcnq1* region was linked with its high pairing frequency. To this end, we analysed two mouse mutants in which imprinting of the *Kcnq1* region is perturbed: One mutant allele carries a polyadenylation cassette which truncates the non-coding RNA Kcnq1ot1. Paternal transmission of this truncation results in derepression of silenced genes in the placenta, and in many embryonic tissues [23]. Surprisingly, cells devoid of repressive Kcnq1ot1 RNA, which therefore feature biallelic expression in the region, still pair with the same frequency as their wildtype counterparts (Fig. 4B). The second mutant allele carries a deletion of the imprinting control region KvDMR1 which not only harbours the promoter for the non-coding RNA Kcnq1ot but also mechanistically important CTCF binding sites [24]. Deletion of KvDMR1 on the paternal allele results in complete loss of imprinting in the region [25]. Again, foetal liver cells carrying the KvDMR1 deletion display the same pairing frequency as wildtype cells (Fig. 4B). We next asked if homologous pairing at the Kcnq1 locus required both maternal and paternal genomes by assessing monoparental ES cell lines. Consistently, no significant differences in pairing frequencies were observed between wildtype, parthenogenetic and androgenetic ES cells (Fig. 4B). Taken together, loss of imprinting in the *Kcnq1* region has no effect on the frequency of homologous pairing.

To determine if homologous pairing was a common feature of imprinted regions we analysed several prominent imprinted loci by 3D DNA FISH (probes around *Ube3a*, *Igf2r*, *Mcts2*, *Dlk1*). In ES cells (Fig. 4C) and foetal liver cells (Fig. S3), none of the regions showed the high pairing frequency that was observed for the *Kcnq1* imprinting cluster on distal chromosome 7. In fact, there was no significant difference in pairing frequency between the group of imprinted regions and a group of non-imprinted control regions (Fig. 4C). In conclusion, we did not find evidence that a high frequency of homologous pairing was generally linked with imprinting or a monoallelic expression state.

### Pairing Frequency is Dependent on Chromosomal Location

In contrast to other analysed imprinted domains the *Kcnq1* locus lies close to the telomere. Telomeres from different chromosomes are known to cluster in interphase nuclei which may be involved in maintaining chromosome positional stability [26]. We placed DNA FISH probes over unique sequence close to the telomeres of chromosomes 4, 5 and 7. All of these showed a high pairing frequency which was significantly different from regions located more centrally (Fig. 4C). This confirms that homologous pairing frequency is dependent on chromosomal location [10].

### Pairing is not Linked with Repair of Double Strand Breaks

We next explored the possibility that the DNA repair machinery could bring homologous regions together. Double strand breaks (DSBs) are a common phenomenon which constantly jeopardises genomic integrity, and homologous recombination (HR) is a major repair pathway rescuing these lesions. In both yeast and mammals, sister chromatids are preferred partners for HR but DSBs can also be efficiently repaired between homologous chromosomes [27]–[30]. We therefore explored the possibility that the homologous pairing we observed was caused by HR repair. We performed DNA immuno FISH with antibodies against two markers of DSBs (γH2AX and p53bp1), but did not observe colocalisation with paired FISH signals (Fig. S4). Similarly, we did not find colocalisation with markers for repair by HR (Rad51 and Rad52). If homologous pairing was caused by DSB repair between non-sister chromosomes it should happen more often during G1 phase when no sister chromatid is available. We therefore assessed at which cell cycle stage pairing events took place. ES cells were enriched by FACS for G1, S and G2 phases according to their DNA content and analysed by DNA FISH (Fig. S5). For both probes, there were seemingly no major differences between cell cycle stages. Thus, we conclude that homologous recombination does not underlie the observed pairing.

### Pairing is Linked with Active Transcription

**Figure 5:**
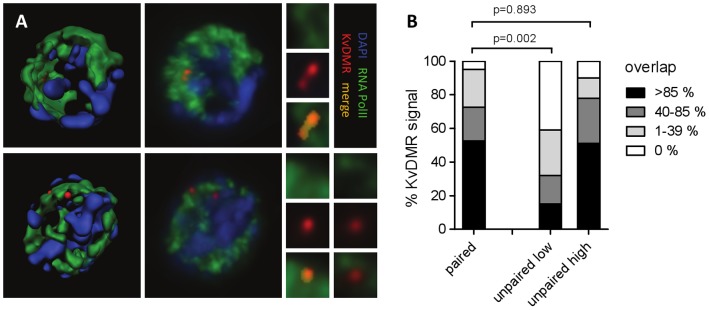
Pairing events occur in regions of active transcription. **A**) 3D representations of image stacks from E13.5 liver cells. Red: KvDMR DNA FISH signals, green: immunostaining for elongating RNA polymerase II, blue: DAPI counterstain. Top panels show a nucleus with paired signals, bottom panels show a nucleus with unpaired signals. Left panels: 3D rendered reconstruction of immuno-FISH data, middle panels: corresponding unrendered images, right panels: blow-up of DNA FISH signals and their colocalisation with RNA PolII immunostain. At this resolution regions of high PolII activity can be clearly defined but do not present as discrete transcription factories. **B**) Quantification of KvDMR signal overlapped by RNA PolII signal. Differences were assessed by t-test. For unpaired alleles, one is generally located in areas rich in RNA PolII (unpaired high) while the other one is mostly found in areas devoid of RNA PolII (unpaired low). Paired alleles are nearly always found in regions of active RNA PolII.

It has previously been shown that transcription can reposition genes and mediate preferential co-associations between chromosomal regions [21]. We therefore assessed if pairing was linked with the expression state of a region. At the *Kcnq1* locus, the paternal allele is transcriptionally silenced while a number of protein coding genes are expressed from the maternal allele. It has been shown by RNA immuno FISH that the active maternal allele colocalises with regions of high RNA polymerase II (RNA PolII) concentration while the paternal allele does not [17]. Accordingly, by DNA immuno FISH we generally find only one allele covered by signal for active RNA PolII (Fig. 5). Strikingly, when KvDMR FISH signals were paired, they both colocalised with RNA PolII demonstrating that pairing events occur in regions of active transcription (Fig. 5). We then assessed a link between pairing frequency of regions analysed by DNA FISH and their expression level (published RNA-Seq data, [31]). Overall, we found a significant correlation between expression and pairing frequency (r = 0.62, p = 0.01, Fig. S6A) but not between gene density and pairing frequency (r = 0.30, p = 0.26, Fig. S6B) indicating that active transcription is a key factor for pairing. Interestingly, the −2 Mb probe which shows the highest pairing frequency in the analysis lies within a gene dense region that is highly transcribed, but not known to be monoallelically expressed (Fig. S6).

## Discussion

We have demonstrated for the first time by allele specific 4C-Seq and by extensive DNA FISH analysis that many loci pair with their homologous allele. Pairing is not limited to regions of mono-allelic expression, involves larger chromosomal regions and brings the two homologous chromosomes into close proximity. While pairing events did not coincide with DNA repair, they took place at sites of ongoing transcription.

Homologous pairing has been implicated in the establishment of mono-allelic silencing of the X chromosome. Indeed, during a period of high chromatin mobility, the two X inactivation centres ‘kiss’ which is followed by transient downregulation of *Tsix* on one allele, thereby creating a window of opportunity for mono-allelic expression of *Xist* [32]. Pairing of homologous alleles is also observed at immunoglobulin loci. One of the paired alleles undergoes RAG mediated cleavage while the other unrearranged allele becomes associated with pericentromeric heterochromatin [4]. These two functionally different examples of mono-allelic expression have in common that one of two equivalent genomic copies is chosen at random for expression. This choice requires some kind of *trans*-allelic cross talk to ensure that one but only one allele gets inactivated. However, for imprinted regions the situation is different. Here, each allele comes in pre-marked and there is no immediate requirement for communication between homologous alleles. Nevertheless, short interallelic distances were observed in late S phase for the Prader-Willi/Angelman imprinted region in human [5], although this effect was argued by others to be due to the presence of a nucleolus organising region on the same chromosome [33]. Here we report high pairing frequency for the *Kcnq1* and adjacent *Igf2/H19* clusters in the mouse, but not for a number of other imprinted clusters. Pairing at distal chromosome 7 was not limited to the imprinted region, and in fact loss of imprinting did not change pairing frequency. Thus, we conclude that homologous pairing is not a general feature of mono-allelically expressed regions. However, this does not preclude an involvement of pairing and *trans*-allelic effects on the regulation of imprinted regions. In fact, it was shown that introducing a third copy of human chromosome 15 disrupted pairing and affected gene expression at the Prader-Willi/Angelman region [34]. Speculatively, at the large *Kcnq1* domain which is silenced by a coating RNA, homologous pairing might be involved in the escape of imprinting of several interspersed biallelically expressed genes, especially as we find that pairing is associated with transcription.

Homologous pairing has also been speculated to be linked with DNA repair. The genome is constantly challenged by double strand breaks (DSB) brought about by internal or external chemical insults or the collapse of stalled replication forks (for review see [35]). These lesions can either be repaired by non-homologous end joining (NHEJ) or homologous recombination (HR). Which repair pathway is used depends on the organism and what caused the double strand break. While HR predominates in yeast, NHEJ plays a more important role in vertebrates. Still, in mammals HR is a common mechanism to repair replication induced damage after fork collapse which leaves a single double strand end. In this scenario the sister chromatid can be used as a template for strand invasion and restart of replication, a process that is helped by sister chromatid cohesion [36]. While it can be envisioned that more severe replication blocks may be repaired by HR involving both homologues, we did not find any evidence that links the homologous pairing described here with DSB or HR repair.

A number of recent genome-wide interaction studies in human cells have demonstrated the presence of topologically distinct active and repressive compartments, with *trans* associations happening preferentially between transcriptionally active regions [37]–[39]. Moreover, a high frequency of *trans* contacts correlated well with the region’s distance to the edge of the chromosome territory [39]. As these studies were not performed in an allele-specific manner, no information about homologous contacts can be drawn. However, it seems likely that for a given region the criteria for a high potential of *trans* interactions, namely transcriptional activity and location close to the edge of the chromosome territory, will also apply to homologous associations. In line with our results, this suggests that not transcription of individual genes but large-scale active features of a region determine a regions propensity to form homologous and non-homologous associations.

Although evidence about regional pairing of homologous chromosomes has increased over recent years, it still remains largely unclear how the two homology partners find each other in the crowded nucleus [40]. In one model, transcription is the driving force: Transcribed genes are located in transcription factories, organising the linear sequence into nodes and intervening loops. By existence of specialised transcription factories a chromosomal transcription signature is created. Because homologues share the same signature, contact at one node increases the probability of larger regions coming together [41]. Indeed, it was recently reported that pairing of the Prader-Willi/Angelman region was reduced upon inhibition of transcription [34]. Our results that pairing frequency is correlated with expression, and that pairing events are located in regions of high RNA PolII activity are in line with this hypothesis. More speculatively, our observation that chromosome territories of paired KvDMR alleles can either be touching at the ends, or be partially or fully aligned, might suggest that once homologous contact has been established in one region, chromosomes have the potential to progressively button up along their whole length.

The data presented here suggest that the frequency by which homologous regions pair is determined by several factors, of which we have identified chromosomal position and transcription, with transcription potentially being the driving force of bringing the two homologous together. This could provide the cell with the potential for another layer of regulation: exchange of information in *trans*. Interestingly, homologous *trans* effects have been reported for multiple loci including imprinted regions. Several studies report introducing mutations into one of the alleles of either the *Igf2*, *Rasgrf1* or Prader-Willi/Angelman region and finding an unexpected effect on expression of the second allele [7], [8], [42]–[44], suggesting that regulatory elements might be functioning in *trans* to enhance or supress transcription. Cross-talk is however not limited to transcriptional regulation but has also been shown to affect allelic methylation. Targeting of the unmethylated paternal *Snrpn* gene in ES cells was frequently associated with full or partial loss of methylation on the maternal allele [42]. Interestingly, allelic methylation was stable when the targeting construct was integrated at heterologous loci, suggesting that both homologues were required to observe a methylation effect in *trans*. Similarly, deletion of the unmethylated maternal *H19* gene led to reduced methylation of the paternal *Igf2* allele [45]. Vice versa, a mutant *Rasgrf1* allele not only attracted methylation to the affected paternal allele, but also in *trans* to the maternal allele [46]. This methylation mark was stable through meiosis and therefore resembles paramutation. Notably, all of the above examples involve imprinted loci. However, only imprinted loci are routinely analysed in an allele specific manner and other *trans* effects might have been missed. Indeed, plasmid DNA containing the beta-globin gene has been shown to physically pair with the homologous region and to transinduce transcription of nearby sequences [47]. In contrast, transactivation was not observed between the beta-globin LCR and its target gene when integrated into the same ectopic site on different chromosomes [22]. This suggests that while pairing events do not necessarily lead to a change in transcriptional output, they have the potential to do so in certain situations.

Taken together, we propose a model in which not the expression state of individual genes but rather the transcriptional signature of large chromosomal domains can bring homologous regions together. Since global chromosomal movements are constrained this might only be possible in a subset of cells which feature a permissive subnuclear arrangement of chromosomes after the last mitosis. Transient allelic interactions in paired regions could then be stabilised to become functionally relevant. Such close proximity could open up the possibility of allelic cross-talk and transcriptional regulation in *trans*, which may in certain circumstances affect normal development and the manifestation of genetic susceptibility to diseases [48], [49].

## Materials and Methods

### Mouse Strains and Cell Lines

All experimental procedures were conducted under licences by the Home Office (UK) in accordance with the Animals (Scientific Procedures) Act 1986. We used C57BL6/JOlaHsd or SD7 as wild-type strains. SD7 contains the distal region of *Mus spretus* chromosome 7 backcrossed into the F1 (C57BL/6J/CBA/Ca), which provides SNPs to distinguish between parental alleles. ES cell genotypes were either C57BL6/JOlaHsd or C57BL6 x SD7. The hybrid ES cell line C57BL6×SD7 carries allele specific information and was newly derived using a modification of a protocol previously described [50]. For details on derivation and characterisation of this ES cell line see File S1.

### 3C and Allele Specific Linear 4C-Seq

3C was performed as described [51]. 3C material was assessed for digestion efficiency and a number of reference *cis* and *trans* interactions. The linear 4C-Seq protocol was adapted from [21]. For details on the linear 4C-Seq assay design, method, sequencing and downstream analysis see File S2.

### 3D DNA FISH, 3D Immuno FISH and Chromosome Painting

DNA FISH probes were directly labelled essentially as described [52]. Briefly, BAC probes (see Table S1) were subjected to nick-translation with 10 U DNA Polymerase I (New England Biolabs) and an individually determined concentration of DNaseI (Roche) in the presence of 60 µM aminoallyl-dUTP (Ambion) at 16°C until most fragments were 200–800 bp in size. DNA was purified by Qiagen PCR purification and EtOH precipitation. Fluorophores (Alexa Fluor 488, 555 or 647) were chemically coupled to aminoallyl-modified DNA using Alexa Fluor reactive dyes (Molecular Probes). One aliquot of reactive dye was used to label up to three probes.

Foetal liver cells settled on poly-L-lysine coated slides in 2 min. ES cells attached to poly-L-lysine coated slides within 3 h in complete medium in a humidified incubator. 3D DNA FISH was performed as described in [53] with the following modifications: After freeze-thawing, cells were incubated twice in PBS (5 min), 0.1 M HCl (30 min), PBS (5 min) and further permeabilised in 0.5% saponin, 0.5% triton X-100 in PBS for 30 min. Slides were equilibrated in 50% formamide/2X SSC for 10 min. Probe mixes (10–50 ng labelled probe, 6 µg C_0_t1 DNA, 10 µg salmon sperm DNA in 50% formamide/10% dextran sulphate/1xSSC) were applied to cells using cover slips sealed on with rubber cement. Samples were denatured at 78°C for 2 min and incubated at 37°C over night. For chromosome painting, directly labelled ready-to-use XMP XCyting mouse chromosome paints (Metasystems) were mixed with precipitated FISH probes. After probe hybridisation, slides were washed in 50% formamide/2×SSC (45°C, 15 min), 0.2×SSC (63°C, 15 min), 2×SSC (45°C, 5 min), 2×SSC (RT, 5 min), PBS (RT, 5 min). For immunostaining, slides were blocked in 3% BSA, 0.05% azide, 0.1% Tween-20 in PBS for 30 min, and incubated for 1 h with primary antibody in blocking solution (for antibodies used see Table S2). Slides were washed twice in 0.2% BSA, 0.1% Tween-20 in PBS and incubated for 30 min with fluorescently labelled secondary antibody in blocking solution before three 10 min washes in 0.2% BSA, 0.1% Tween-20 in PBS; 0.1% Tween-20 in PBS and PBS. Nuclei were stained with DAPI (1∶1000) for 2 min in 2×SSC and washed in PBS (5 min). Coverslips were mounted in Vectashield (Vector Labs) or SlowFade Gold (Molecular Probes).

### Microscopy and Image Analysis

Automated image capture and analysis was performed using the Metasystems Metafer slide scanning platform in conjuction with a Zeiss Axio Imager Z2 upright microscope using a 100×1.4 NA plan apochromat lens and Metafer4.metacyte (version 3.8) software. All acquired images were post-analysed by eye to identify pairing events. For each biological sample the frequency of pairing events was determined from two sets of 300 imaged nuclei, scored by two different people in a sample blind way. A FISH signal was counted as ‘paired’ if i) the spots were so close together that the MetaCyte software would only recognise one signal but two spots were discernible by eye, ii) the MetaCyte signal annotation was in the middle of the two spots and iii) there were no other signals visible in the nucleus. Since the z-planes of the image stacks are 0.5 µm apart, this was considered the maximal resolution of the analysis. Paired signals are therefore less than 0.5 µm apart.

For DNA immuno FISH, pairing events were identified using automated image capture followed by manual acquisition of image stacks using ISIS software (Metasystems, version 5.4). For chromosome painting, nuclei were imaged on an Olympus IX81 confocal microscope (FV1000) using a 60×1.35 NA plan apochromat lens and Olympus fluoview software (version 3.0). Deconvolution of captured image stacks was performed with Huygens Professional software (Scientific Volume Imaging, version 4.1). Imaris software (Bitplane, version 7.3) was used for image analysis and 3D modelling.

### Simulated FISH

A computational model was developed in R to simulate the position of two FISH signals inside the nucleus which display preferential radial positions. The constraint on radial distribution is attained by setting up two exclusion limits for each allele in the nucleus: a central exclusion limit (minimum distance from the nuclear centre) and a peripheric exclusion limit (maximum distance from the centre). For each simulated signal, the limits are randomly chosen based on a normal distribution whose mean and standard deviation are input by the user. By adjusting the input variables the radial distribution of simulated signals is matched to the radial distribution of the respective measured FISH signals. As an output, the model displays the distance between two simulated FISH signals.

### Enrichment for Cell Cycle Stages

ES cells were fixed in 4% PFA in PBS for 10 min. Dye Cycle Violet staining was performed according to instructions of the manufacturer (Vybrant Dye Cycle Violet stain, Molecular Probes). Briefly, fixed cells were washed in 0.1 M Tris-Cl (pH 7.4) and then incubated with 10 µM Dye Cycle Violet in 0.1 M Tris-Cl for 30 min at 37°C. Samples were FACS sorted on a BD FACS Aria3 using violet 405 nm excitation with a 450/40 nm bandpass filter. Cell fractions were attached to slides using a cytospin (300 rpm, 3 min). DNA FISH was performed as normal.

## Supporting Information

Figure S1 **Radial distributions of DNA FISH signals and simulated counterparts.** Radial distances for KvDMR (**A**) and myc (**B**) DNA FISH signals from E13.5 liver nuclei were determined and plotted as a histogram (grey bars, n = 600). Radial distances >1 can occur if the nucleus is not a perfect sphere. For both probes, FISH signals show a highly non-random radial distribution and thus cannot be compared to a simulated data set in which signals are placed in a sphere at random. Therefore, locations of signals were simulated to reflect the radial distribution of the respective probe (blue line, n = 600), but are otherwise random. The distance between pairs of simulated signals was then calculated and is plotted in Fig. 2D. **C**) Histogram of interallelic distances of KvDMR and myc signals, measured and simulated. Interallelic distances for 600 nuclei were grouped into four equal bins (bin width  = 0.7r). Bin centres are indicated (0, 0.7, 1.4 and 2.1r). The histogram displays the same data that is shown as Tukey box-whisker plots in Fig. 2D to illustrate the skewing towards very short distances for the measured KvDMR signals.
(TIF)

Figure S2 **Pairing of the KvDMR region in ES cells. A**) Pairing frequency of the KvDMR and myc regions in ES cells. Each dot represents one sample from one cell passage, and for each sample 300 nuclei were counted in four technical replicates. Differences were assessed by unpaired t-test, ns: not significant, *: p<0.05, ***: p<0.001. KvDMR signals show higher pairing frequency than myc signals. **B**) Distances between homologous alleles in E13.5 liver represented as Tukey box-whisker plots (n = 600). Differences were assessed by one-way ANOVA with Bonferroni’s multiple comparison post test. Simulated: a group of spots displaying the same radial distributions as KvDMR and myc FISH signals, respectively, were placed into a sphere at random and their interallelic distances determined. While interallelic distances between myc signals show a distribution expected from their radial positions (no difference between sample and simulated), KvDMR signals are significantly closer together than expected (difference between sample and simulated, p<0.001). Also, KvDMR signals are generally closer together than myc signals.
(TIF)

Figure S3 **Pairing frequencies of imprinted regions in ES cells.** Each dot represents the mean of three to four samples taken from different passages with the pairing frequency determined for each sample in four technical replicates of 300 nuclei. Whiskers represent standard deviation. Background shading indicates high pairing frequency (dark grey, above 3.5%), medium (light grey, 2.5–3.5%) and low pairing frequency (yellow, below 2.5%).
(TIF)

Figure S4 **Paired KvDMR FISH signals do not colocalise with markers for DNA double strand breaks or repair by homologous recombination.** 3D representations of image stacks from E13.5 liver cells. Red: KvDMR DNA FISH signals, green: immunostaining for markers of DNA double strand breaks (γH2AX (**A**), p53bp1 (**B**)), or markers for homologous recombination (Rad51 (**C**), Rad52 (**D**)), blue: DAPI counterstain. Numbers for analysed pairing events are indicated. No overlap of immuno and FISH signals was observed.
(TIF)

Figure S5 **Pairing is not specific to a certain cell cycle stage. A**) Histogram of PFA fixed ES cells stained with DyeCycle Violet showing DNA content distribution with peaks for G1 and G2 phase cells. The high proportion of S-phase cells is typical for ES cells. **B**) Pairing frequencies for subpopulations of cells FACS sorted for cell cycle stages. Red dots: KvDMR signals, green squares: myc signals. Each dot represents the mean of three samples taken from different passages with the pairing frequency determined for each sample in two to four technical replicates of 300 nuclei.
(TIF)

Figure S6 **Pairing frequency is correlated with expression. A**) RNA Seq reads (ES cell dataset from [31]) were counted per DNA FISH probe and plotted against the pairing frequency in ES cells. Pearson correlation analysis shows a significant positive correlation (r = 0.62, p = 0.01). **B**) The percentage of DNA FISH probe covered by genic sequence was determined using the Ensembl annotation and plotted against the pairing frequency in ES cells. No significant Pearson correlation is observed (r = 0.30, p = 0.26).
(TIF)

Table S1 **BACs labelled to create FISH probes.**
(DOC)

Table S2 **Antibodies.**
(DOC)

File S1 **Derivation and characterisation of the ES cell line B6xSD7.**
(DOC)

File S2 **Details of the linear 4C-Seq analysis.**
(DOC)

Movie S1 **Regional pairing brings chromosome domains close together.**
(MOV)
