# Supplementary figures and images for: Pairing of Homologous Regions in the Mouse Genome Is Associated with Transcription but Not Imprinting Status
Source: PLoS One. 2012 Jul 3;7(7):e38983. doi: 10.1371/journal.pone.0038983 (PMC3389011; doi:10.1371/journal.pone.0038983)

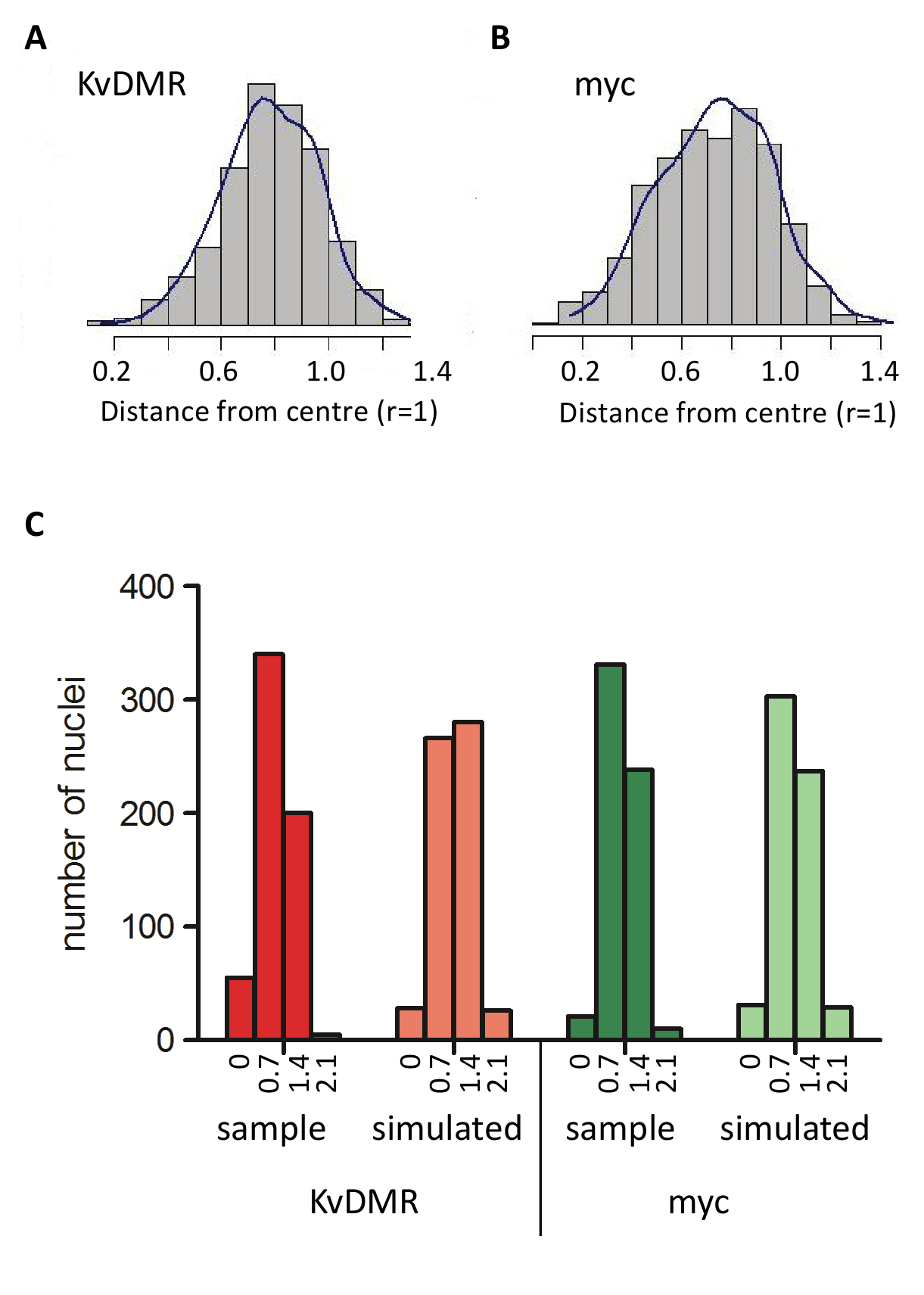

Supplement: Figure S1 — Radial distributions of DNA FISH signals and simulated counterparts. Radial distances for KvDMR (A) and myc (B) DNA FISH signals from E13.5 liver nuclei were determined and plotted as a histogram (grey bars, n = 600). Radial distances >1 can occur if the nucleus is not a perfect sphere. For both probes, FISH signals show a highly non-random radial distribution and thus cannot be compared to a simulated data set in which signals are placed in a sphere at random. Therefore, locations of signals were simulated to reflect the radial distribution of the respective probe (blue line, n = 600), but are otherwise random. The distance between pairs of simulated signals was then calculated and is plotted in Fig. 2D. C) Histogram of interallelic distances of KvDMR and myc signals, measured and simulated. Interallelic distances for 600 nuclei were grouped into four equal bins (bin width = 0.7r). Bin centres are indicated (0, 0.7, 1.4 and 2.1r). The histogram displays the same data that is shown as Tukey box-whisker plots in Fig. 2D to illustrate the skewing towards very short distances for the measured KvDMR signals. (TIF) [file pone.0038983.s001.tif]

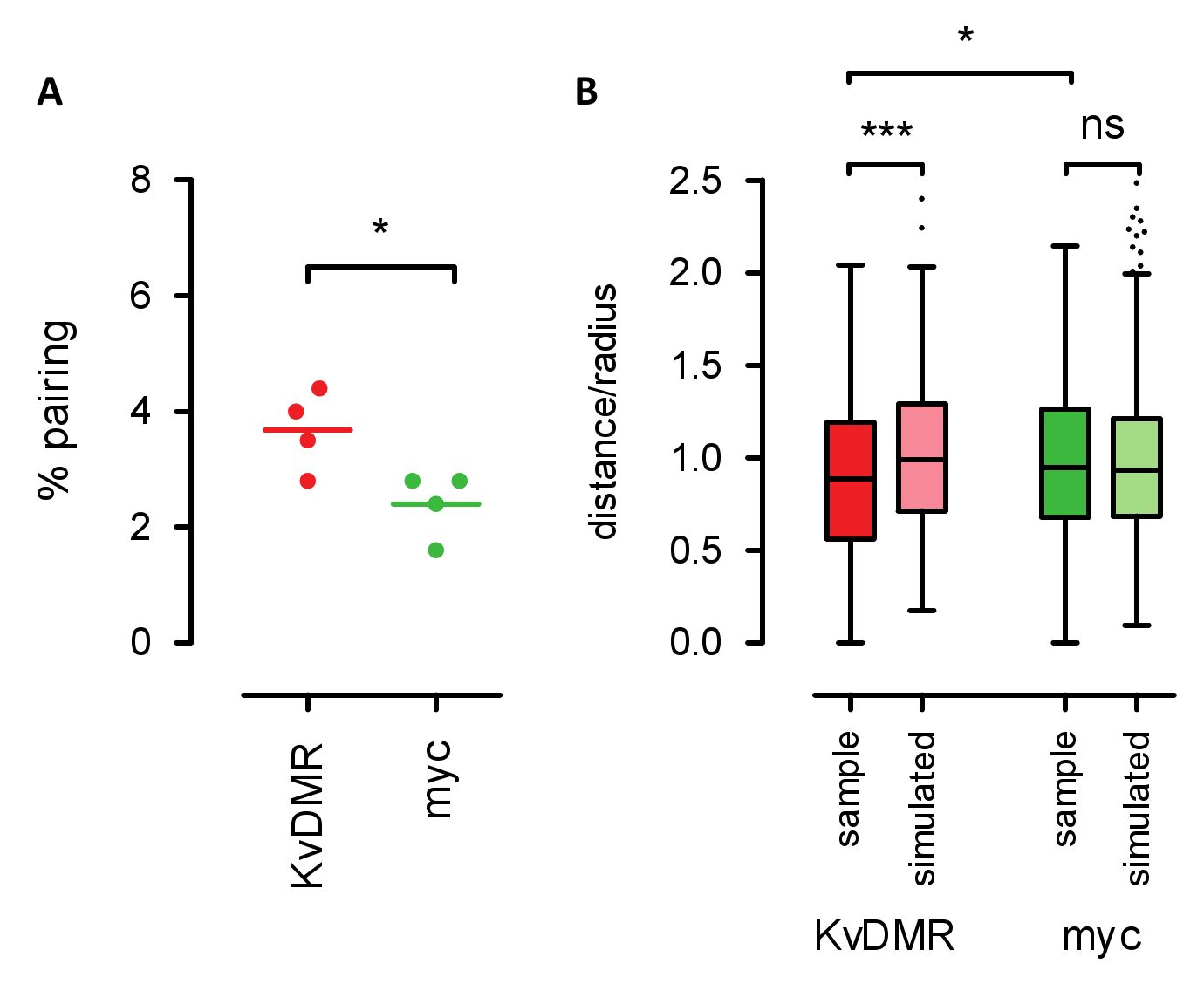

Supplement: Figure S2 — Pairing of the KvDMR region in ES cells. A) Pairing frequency of the KvDMR and myc regions in ES cells. Each dot represents one sample from one cell passage, and for each sample 300 nuclei were counted in four technical replicates. Differences were assessed by unpaired t-test, ns: not significant, *: p<0.05, ***: p<0.001. KvDMR signals show higher pairing frequency than myc signals. B) Distances between homologous alleles in E13.5 liver represented as Tukey box-whisker plots (n = 600). Differences were assessed by one-way ANOVA with Bonferroni’s multiple comparison post test. Simulated: a group of spots displaying the same radial distributions as KvDMR and myc FISH signals, respectively, were placed into a sphere at random and their interallelic distances determined. While interallelic distances between myc signals show a distribution expected from their radial positions (no difference between sample and simulated), KvDMR signals are significantly closer together than expected (difference between sample and simulated, p<0.001). Also, KvDMR signals are generally closer together than myc signals. (TIF) [file pone.0038983.s002.tif]

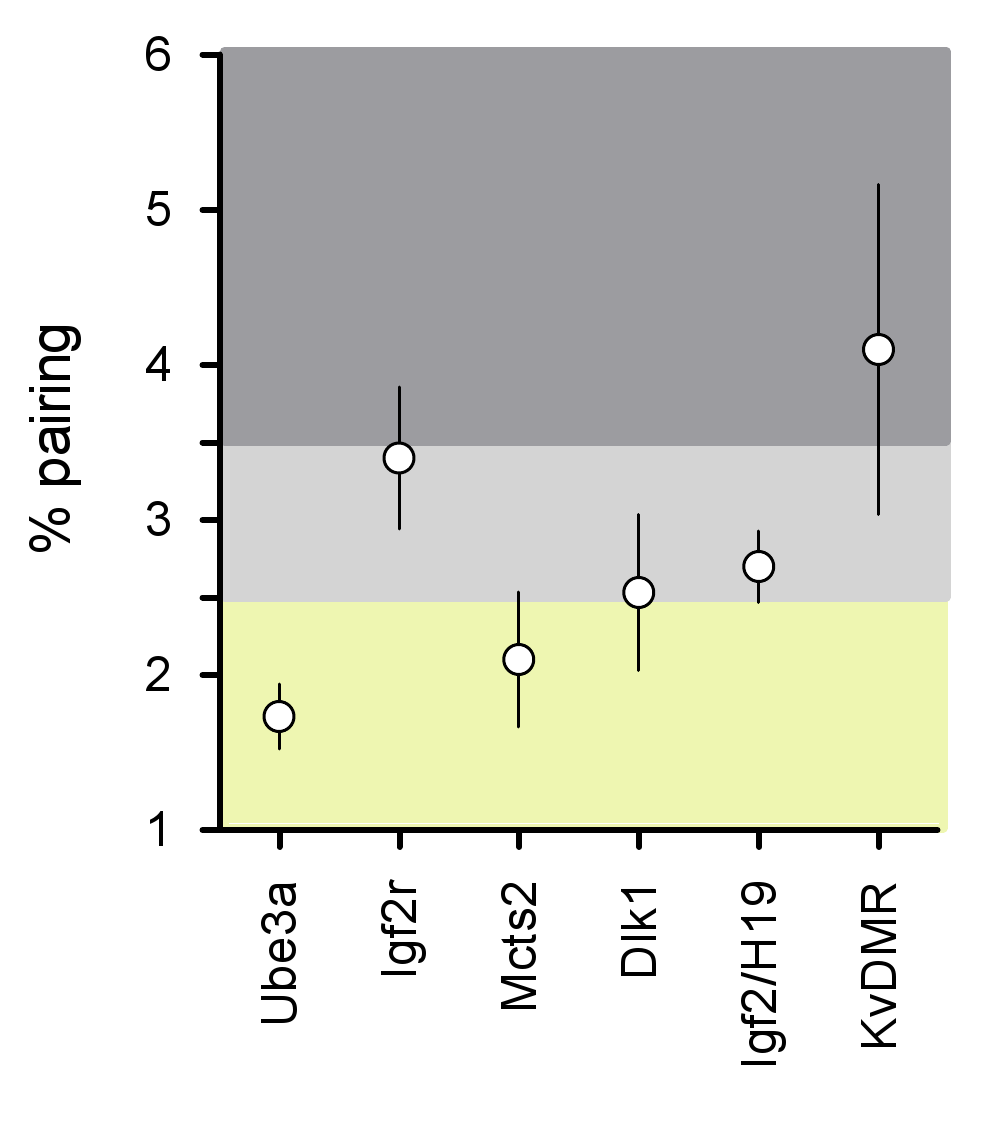

Supplement: Figure S3 — Pairing frequencies of imprinted regions in ES cells. Each dot represents the mean of three to four samples taken from different passages with the pairing frequency determined for each sample in four technical replicates of 300 nuclei. Whiskers represent standard deviation. Background shading indicates high pairing frequency (dark grey, above 3.5%), medium (light grey, 2.5–3.5%) and low pairing frequency (yellow, below 2.5%). (TIF) [file pone.0038983.s003.tif]

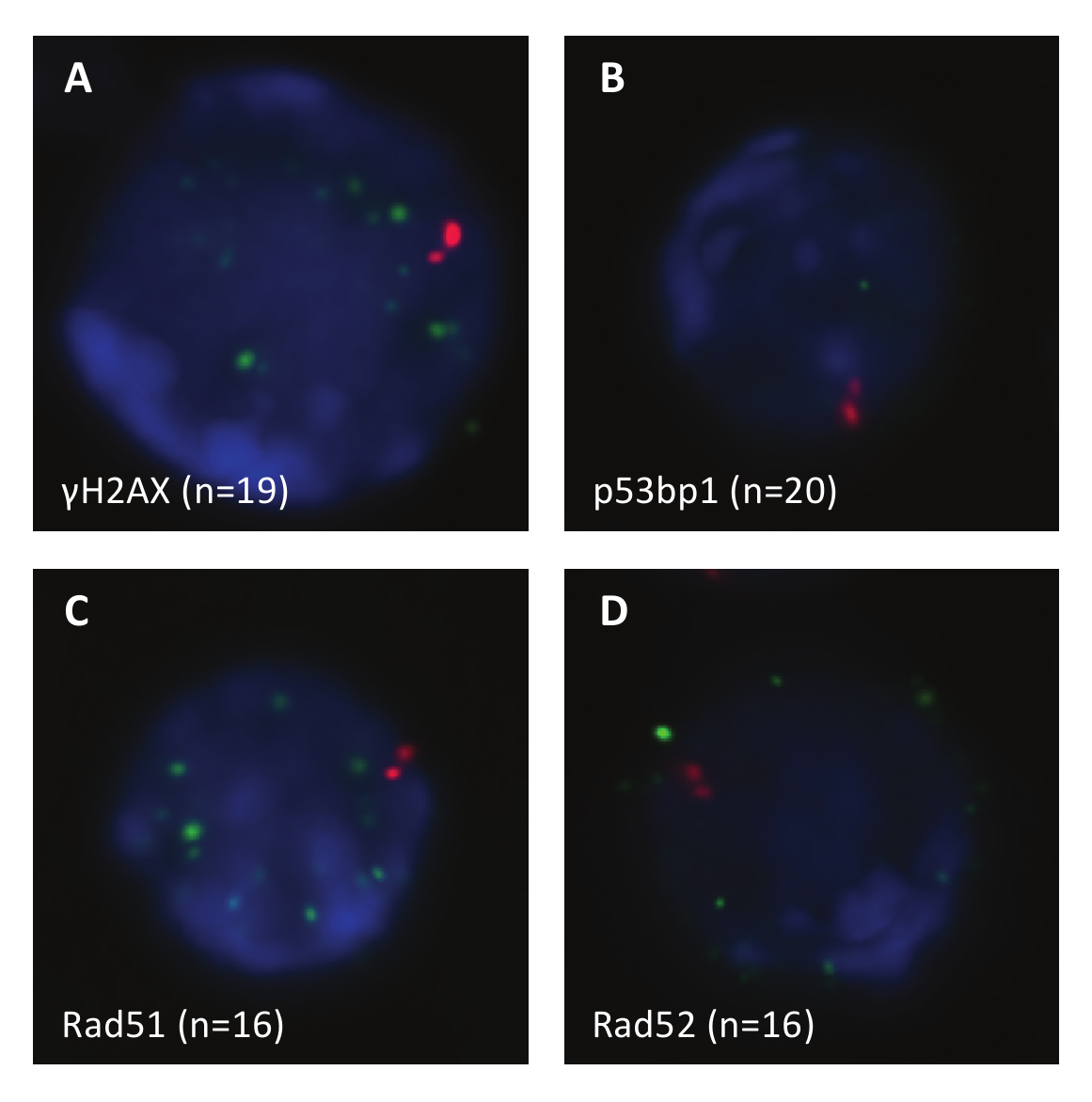

Supplement: Figure S4 — Paired KvDMR FISH signals do not colocalise with markers for DNA double strand breaks or repair by homologous recombination. 3D representations of image stacks from E13.5 liver cells. Red: KvDMR DNA FISH signals, green: immunostaining for markers of DNA double strand breaks (γH2AX (A), p53bp1 (B)), or markers for homologous recombination (Rad51 (C), Rad52 (D)), blue: DAPI counterstain. Numbers for analysed pairing events are indicated. No overlap of immuno and FISH signals was observed. (TIF) [file pone.0038983.s004.tif]

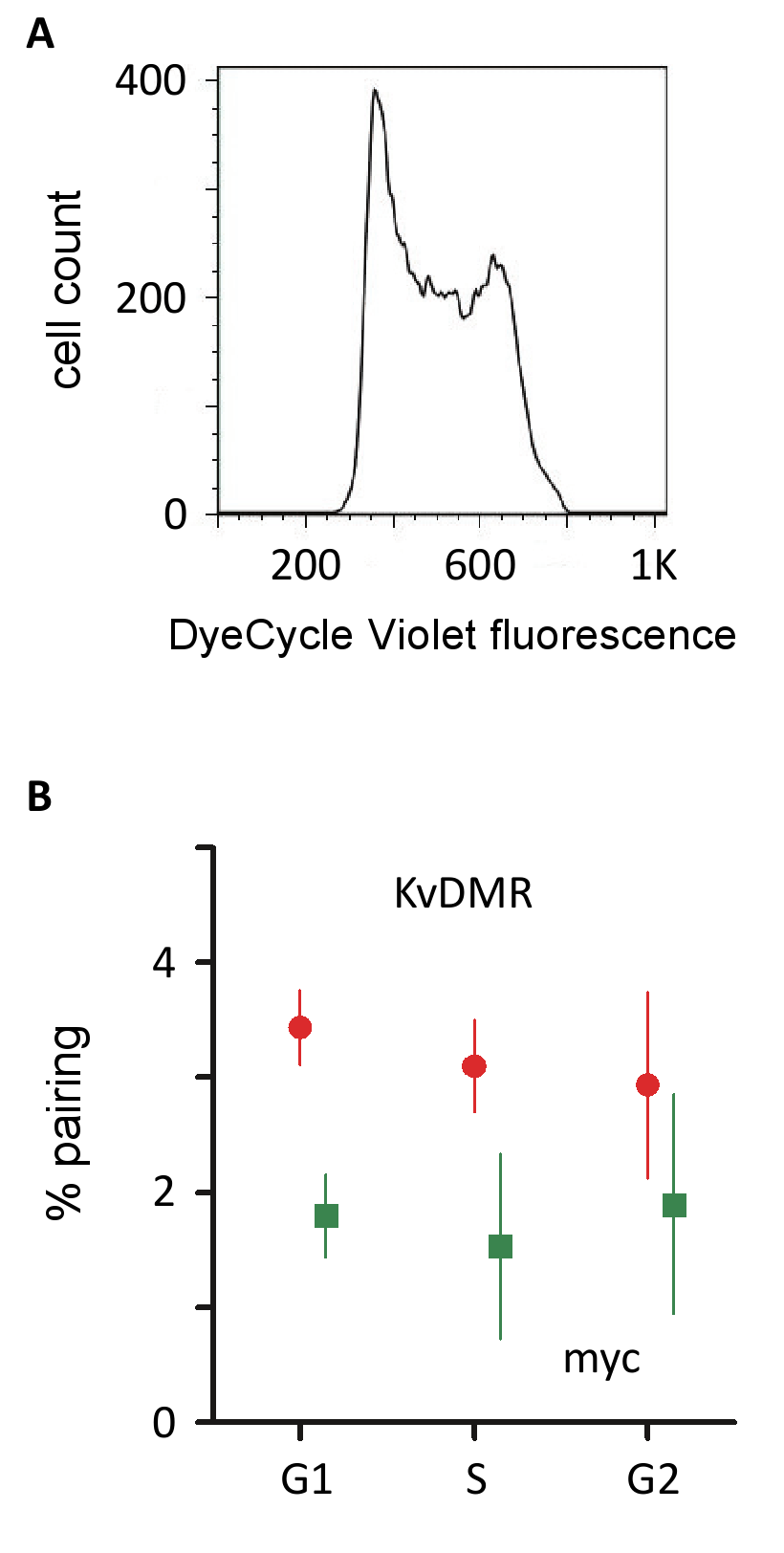

Supplement: Figure S5 — Pairing is not specific to a certain cell cycle stage. A) Histogram of PFA fixed ES cells stained with DyeCycle Violet showing DNA content distribution with peaks for G1 and G2 phase cells. The high proportion of S-phase cells is typical for ES cells. B) Pairing frequencies for subpopulations of cells FACS sorted for cell cycle stages. Red dots: KvDMR signals, green squares: myc signals. Each dot represents the mean of three samples taken from different passages with the pairing frequency determined for each sample in two to four technical replicates of 300 nuclei. (TIF) [file pone.0038983.s005.tif]

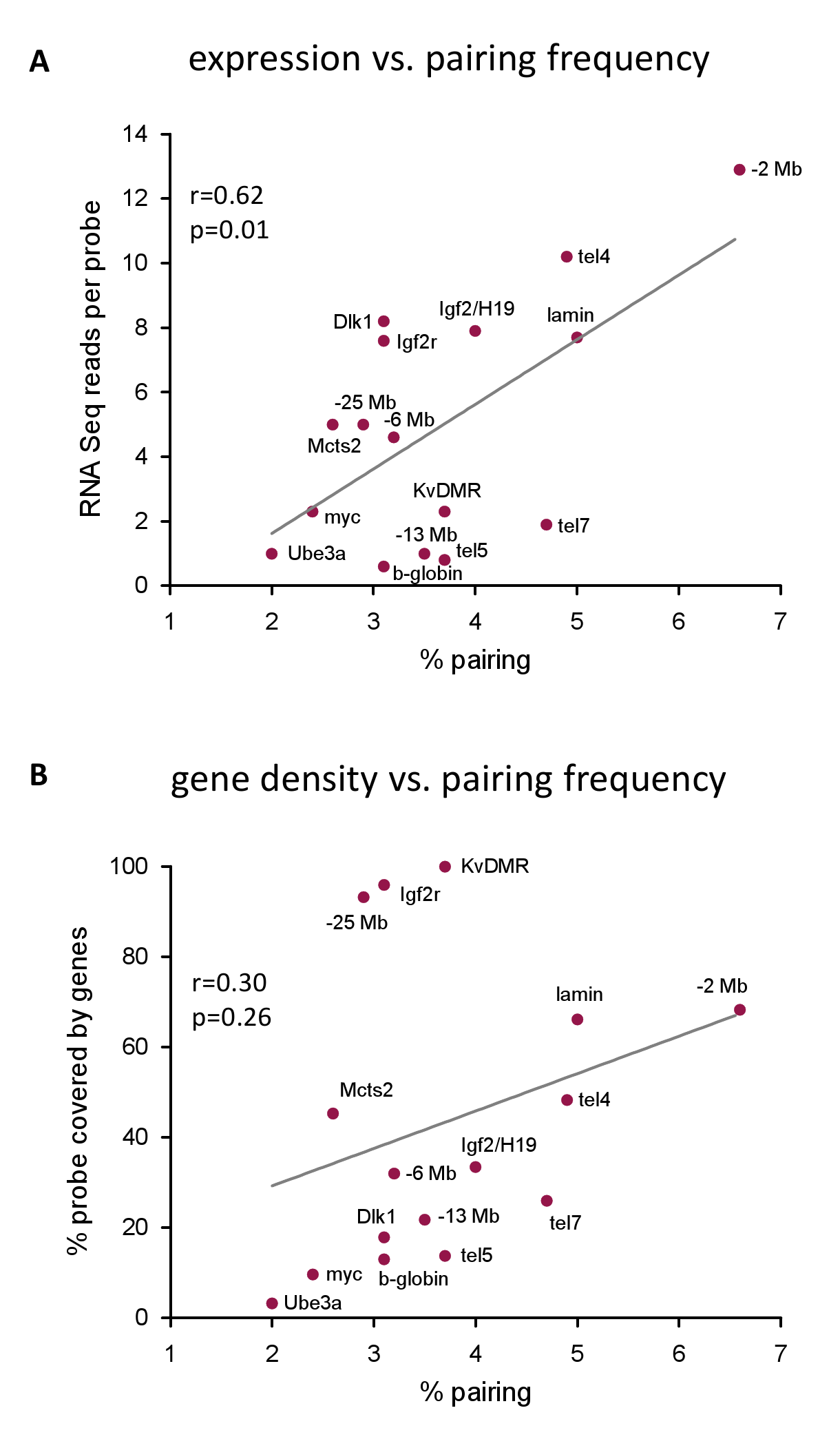

Supplement: Figure S6 — Pairing frequency is correlated with expression. A) RNA Seq reads (ES cell dataset from [31]) were counted per DNA FISH probe and plotted against the pairing frequency in ES cells. Pearson correlation analysis shows a significant positive correlation (r = 0.62, p = 0.01). B) The percentage of DNA FISH probe covered by genic sequence was determined using the Ensembl annotation and plotted against the pairing frequency in ES cells. No significant Pearson correlation is observed (r = 0.30, p = 0.26). (TIF) [file pone.0038983.s006.tif]
