# Supplementary material for: Pairing of Homologous Regions in the Mouse Genome Is Associated with Transcription but Not Imprinting Status
Source: PLoS One. 2012 Jul 3;7(7):e38983. doi: 10.1371/journal.pone.0038983 (PMC3389011; doi:10.1371/journal.pone.0038983)
Supplement: Table S1 — BACs labelled to create FISH probes. (DOC) [file pone.0038983.s007.doc]

**Table S1: BACs labelled to create FISH probes**

| **designation** | **clone** | **size (kb)** | **start** | **end** | **Chr** |
| --- | --- | --- | --- | --- | --- |
| **tel7** | RP24-316N16 | 184.9 | 152331071 | 152515971 | 7 |
| **Osbpl5** | RP23-16K9 | 209.8 | 150833617 | 151043416 | 7 |
| **Cdkn1c** | RP23-124B2 | 270.9 | 150489711 | 150760563 | 7 |
| **Th** | RP23-92I23 | 276.5 | 149852567 | 150129044 | 7 |
| **IC1** | RP23-209O22 | 194.3 | 149703972 | 149898270 | 7 |
| **-2 Mb** | RP23-134J4 | 220.4 | 148440259 | 148660628 | 7 |
| **-6 Mb** | RP23-418L3 | 206.4 | 144556393 | 144762820 | 7 |
| **-13 Mb** | RP24-562C11 | 236.8 | 137120724 | 137357488 | 7 |
| **-25 Mb** | RP24-127G8 | 199.6 | 125952681 | 126152305 | 7 |
| **β-globin** | RP24-344M21 | 190.2 | 110840403 | 111030620 | 7 |
| **Ube3a** | RP23-410L2 | 181.3 | 66573491 | 66754815 | 7 |
| **tel4** | RP23-139J18 | 201.9 | 155358050 | 155559977 | 4 |
| **tel5** | RP23-138J7 | 183.4 | 152259661 | 152443090 | 5 |
| **Gnas** | RP23-312C1 | 166.7 | 174058706 | 174225449 | 2 |
| **Dlk1** | RP23-75I2 | 231.2 | 110582554 | 110813715 | 12 |
| **Mcts2** | RP23-140E20 | 179.7 | 152418503 | 152598209 | 2 |
| **Igf2r** | RP24-545N6 | 161.9 | 12860658 | 13022568 | 17 |
| **myc** | RP23-98-D8 | 234.1 | 61653069 | 61887193 | 15 |
| **KvDMR** | RP23-101N20 | 277.0 | 150331359 | 150608312 | 7 |
| **lamin** | RP23-87N23 | 180.9 | 80280081 | 80460968 | 10 |
