# Supplementary material for: Pairing of Homologous Regions in the Mouse Genome Is Associated with Transcription but Not Imprinting Status
Source: PLoS One. 2012 Jul 3;7(7):e38983. doi: 10.1371/journal.pone.0038983 (PMC3389011; doi:10.1371/journal.pone.0038983)
Supplement: Table S2 — Antibodies. (DOC) [file pone.0038983.s008.doc]

**Table S2: Antibodies**

| **designation** | **manufacturer** | **dilution** |
| --- | --- | --- |
| **Anti-RNA polymerase II, clone CTD4H8** | Upstate | 1:200 |
| **Anti-phospho-Histone H2A.X (Ser139), clone JBW301** | Upstate | 1:400 |
| **Anti-53BP1 (H-300): sc-22760** | Santa Cruz | 1:400 |
| **Anti-Rad51 (Ab1)** | Calbiochem | 1:400 |
| **Anti-Rad52 (H-300): sc-8350** | Santa Cruz | 1:500 |
| **Alexa Fluor 488 donkey anti-mouse IgG (H+L)** | Molecular Probes | 1:1000 |
| **Alexa Fluor 488 donkey anti-rabbit IgG (H+L)** | Molecular Probes | 1:500 |
