# Supplementary material for: Pairing of Homologous Regions in the Mouse Genome Is Associated with Transcription but Not Imprinting Status
Source: PLoS One. 2012 Jul 3;7(7):e38983. doi: 10.1371/journal.pone.0038983 (PMC3389011; doi:10.1371/journal.pone.0038983)

**File S1: Derivation and characterisation of the ES cell line B6xSD7**

*Derivation*

Heterozygous blastocysts from a C57BL6/JOlaHsd x SD7 cross were harvested and treated briefly with acidic tyrode’s solution (Chemicon) solution to dissolve the zona pellucida. ES cells were derived in ES cell media consisting of: 85% DMEM:F12 media (Invitrogen), 20% knockout serum replacer (Invitrogen), 1% L-glutamine (Sigma), 1% Penicillin/Streptomycin (Sigma), 0.1 mM β-mercaptoethanol (Sigma), 10^3^ IU of LIF (Chemicon), supplemented with 50 µM of MEK inhibitor (PD98059, Cell Signaling). Zona-free blastocysts were plated onto γ-irradiated murine embryonic fibroblast coated, pre-gelatinized tissue culture plates. The inner cell mass (ICM) of the outgrowths were picked after 7-14 days, washed briefly in 1X PBS and placed briefly into 0.05% trypsin to dissociate the outgrowth. The ICM outgrowths were then expanded in ES cell media and analysed by immunofluorescence: Samples were fixed in 4% PFA at 4 °C overnight, then permeabilized with 0.5% Tween in 1X PBS for 20 min and blocked with 10% FBS diluted in 0.1% Tween in 1X PBS for 1 h. Mouse anti-Oct4 (Santa Cruz Biotech, sc5279) and rabbit anti-Nanog (2B Scientific, REC-RCAB0001) primary antibodies were diluted at 1:500 in blocking solution and samples were incubated at 4°C rotating overnight. Secondary antibodies were added at 1:300 (Molecular Probes) and samples were incubated for 1 h at room temperature. Finally, samples were washed and covered with 0.1% Tween in 1X PBS containing DAPI Vectashield mounting medium (Vector Labs). Two clones (#5 and #10) displaying typical ES cell morphology and strong immunofluorescent signals for Oct4 and Nanog were chosen for further analysis. Only results for #5 are shown here as this is the clone that has been used for all subsequent experiments.


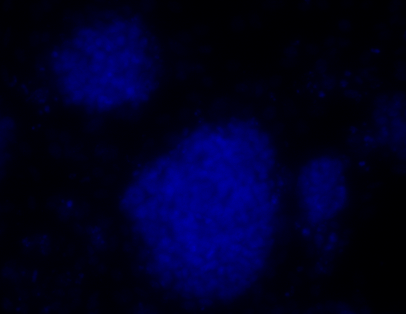

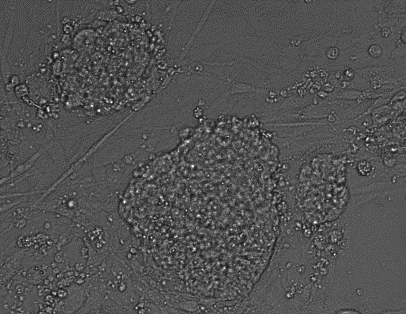

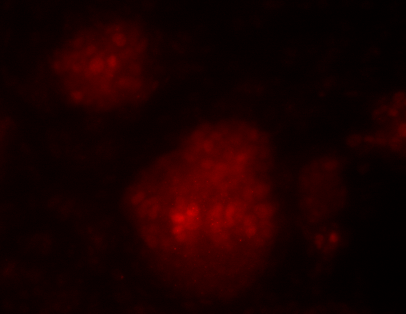

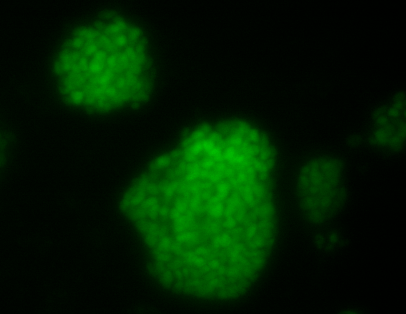


**A**

**B**

**Immunofluorescent staining for B6xSD7 ES cell clone #5**

**A)** Brightfield image of P3 colonies of clone #5, **B)** DAPI staining, **C)** Oct4 immunofluorescence image, **D)** Nanog immunofluorescence image.

**C**

**D**

*Alkaline Phosphatase Assay*

Undifferentiated ES cells express high levels of alkaline phosphatase. Its activity can be visualised by production of dye from a phosphorylated substrate. The Alkaline Phosphatase Detection Kit (Millipore) was used according to instructions from the manufacturer. All colonies showed intense purple staining indicating phosphatase activity while feeder cells remained colourless.


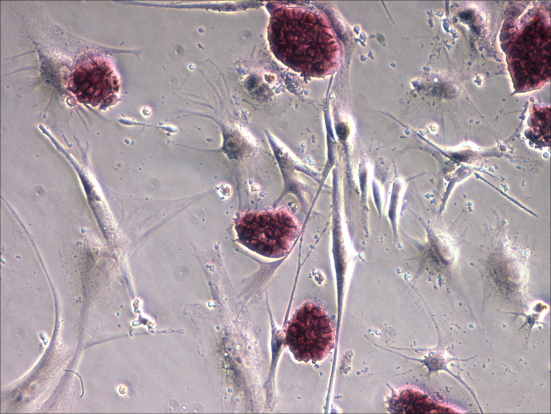

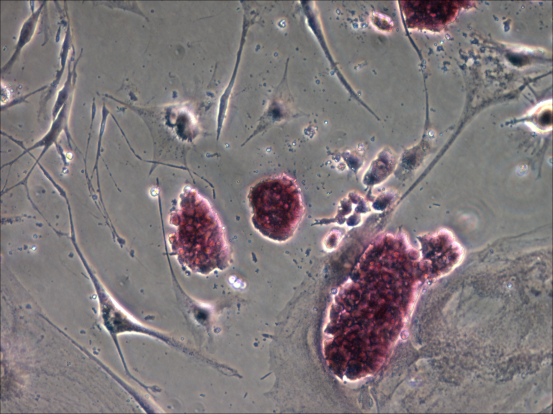


*Expression of pluripotency markers*

RNA was isolated from cell lines using Trizol (Invitrogen) and cDNA was prepared using Superscript III (Invitrogen). RNA from two cell passages of B6xSD7 ES cells was analysed and compared to RNA from an in house B6 ES cell line (TMDB10, derived from C57Bl/6, routinely used for gene targeting) and J1 ES cells (129S4/SvJae). Samples were normalised to J1. Six pluripotency markers were analysed. Atp5b and Hsbcb were included as reference genes to gauge preparation variability between samples.

Pluripotency markers are expressed at different levels between passages and different ES cell lines. The newly derived B6xSD7 ES cell line shows a similar expression profile to B6 ES cells.

**Table of primers:**

| **Gene** | **Primer Designation** | **Sequence** |
| --- | --- | --- |
| **Oct4** | RT-F-Oct4 | CCAATCAGCTTGGGCTAGAG |
|  | RT-R-Oct4 | CCTGGGAAAGGTGTCCCTGTA |
| **Nanog** | RT-F-Nanog | AAGCAGAAGATGCGGACTGT |
|  | RT-R-Nanog | ATCTGCTGGAGGCTGAGGTA |
| **Stella** | Dppa3_FW | TGTCGGTGCTGAAAGACCCTAT |
|  | Dppa3-RV | TCTGAATGGCTCACTGTCCCG |
| **Sall4** | RT-FW-Sall4 | GAGCAGCCTCAGCAGCTACC |
|  | RT-RV-Sall4 | CACCGGTATGGAGTCCTCTGA |
| **Esrrb** | RT-F-Esrrb | AGTACAAGCGACGGCTGGAT |
|  | RT-R-Esrrb | CCTAGTAGATTCGAGACGATCTTAGTCA |
| **Tet1** | Tet1-FW | CCATTCTCACAAGGACATTCACA |
|  | Tet1-RV | GCAGGACGTGGAGTTGTTCA |
| **Atp5b** | Atp5b-1-f | GGCCAAGATGTCCTGCTGTT |
|  | Atp5b-1-r | GCTGGTAGCCTACAGCAGAAGG |
| **Hspcb** | Hspcb-1-f | GCTGGCTGAGGACAAGGAGA |
|  | Hspcb-1-r | CGTCGGTTAGTGGAATCTTCATG |

*Methylation status of the KvDMR1 and H19 DMR*

1.5 ug of genomic DNA isolated by phenol/chloroform extraction was bisulfite converted using the Epitect Kit (Qiagen). DMRs were amplified with the primers shown below using HotStar Taq (Qiagen; 1 ul template, 54 °C annealing, 35 cycles). PCR products were purified by gel extraction (Qiagen MinElute) and cloned into the pGEM-T Easy Vector (Promega). White colonies were resuspended in T_0.1_E and used as a template to amplify the DMR insert with universal primers M13-fw and M13-rev (Sigma). PCR products were sequenced using universal primer T7 or SP6 (Sigma).

| **Region** | **Primer designation** | **Sequence** |
| --- | --- | --- |
| **KvDMR1** | KvDMR-bis-fw | GGTTTTAAGATTATTTTTGTTTTTGTAA |
|  | KvDMR-bis-rev | TTTTCTATTCAACTTAATTCCCAAC |
| **H19 DMR** | H19 dmd 2/3 (seq)F+tag | AGGAAGAGAGGATTTGGTTATAGTTAAATGGATAG |
|  | H19 dmd 2/3 (seq)R | AACATTACAATAATTAAACCCCA |

**Methylation of KvDMR1**

Since the KvDMR1 amplicon contained two indels and two SNPs, allelic origin of the sequence could be determined. Sequences were analysed in Quma (<http://quma.cdb.riken.jp/>). KvDMR1 is completely methylated on the maternal allele while unmethylated on the paternal allele. Methylated CpGs are represented by filled circles, unmethylated CpGs are shown as open circles.

B6 (maternal allele)


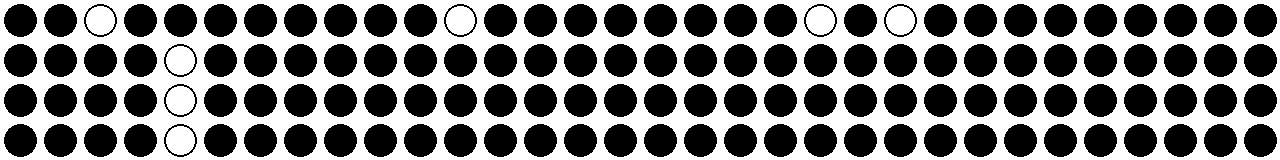


SD7 (paternal allele)


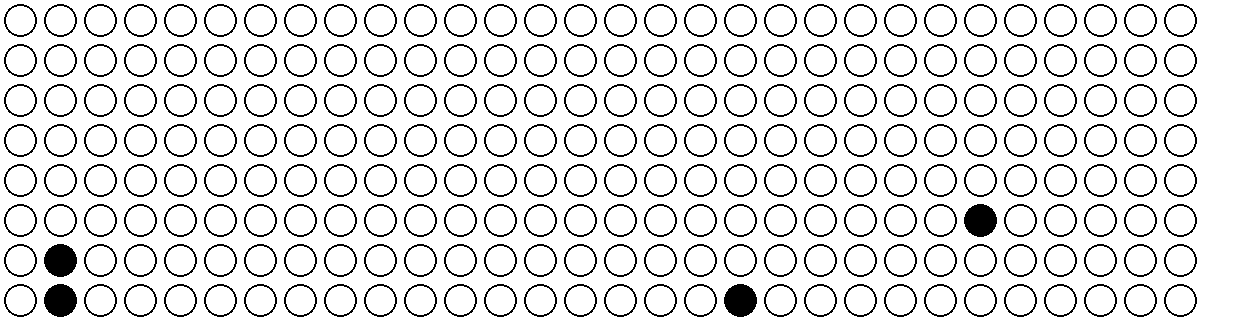


**Methylation of H19 DMR**

The H19 DMR amplicon contained a SNP which was used to determine allelic origin. Analysis was done as described above. The newly derived hybrid ES cell line shows partial methylation on the maternal allele and almost full methylation on the paternal allele. Of note, our in-house B6 ES cell line shows complete methylation in the region, suggesting methylation on both parental alleles.

B6 (maternal allele)


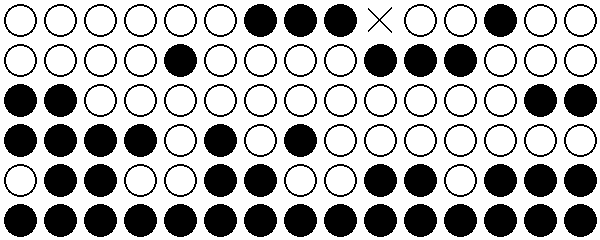


SD7 (paternal allele)


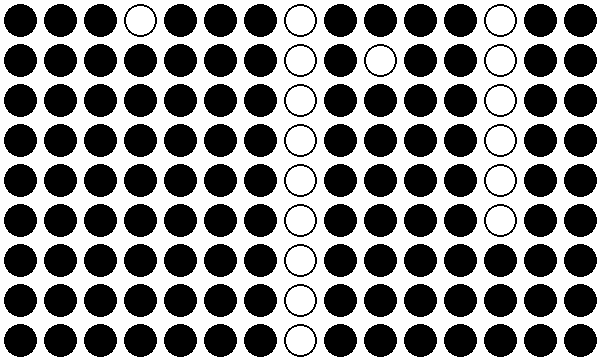

Supplement: File S1 — Derivation and characterisation of the ES cell line B6xSD7. (DOC) [file pone.0038983.s009.doc]
