# Supplementary material for: Pairing of Homologous Regions in the Mouse Genome Is Associated with Transcription but Not Imprinting Status
Source: PLoS One. 2012 Jul 3;7(7):e38983. doi: 10.1371/journal.pone.0038983 (PMC3389011; doi:10.1371/journal.pone.0038983)
Supplement: File S2 — Details of the linear 4C-Seq analysis. (DOC) [file pone.0038983.s010.doc]

**File S2: Details of the linear 4C-Seq analysis**

*Linear 4C-Seq assay design*

3C material was generated using BglII as a restriction enzyme as described in . The bait fragment (Chr7: 150474507-150484686, NCBIM37) encompasses the whole KvDMR1, and was enriched by streptavidin pulldown of a biotinylated primer extension product using primer KvDMR-4C-ext as described in Schoenfelder et al . The 4-cutter Csp6I was used to cut within the prey region and to create uniform ends for adapter ligation. A schematic of the chimeric product and subsequent PCR steps is shown below:

After streptavidin enrichment of 3C fusion products containing the KvDMR bait region, and Csp6I restriction, chimeric products consist of a defined part of the bait region (black) and an unknown prey region (red) ending with a Csp6I site. Standard Illumina adapters are partially double stranded (forked) to create two different ends after amplification with standard Illumina paired-end primers . To mimic this directionality, we designed an adapter representing end 1 of the Illumina adapters and containing a Csp6I compatible overhang. The adapter (purple) was hybridised using oligos Csp6I-ia-oe-for and Csp6I-ia-oe-rev and ligated to the Csp6I restricted pull-down material. To introduce the Illumina adapter end 2 on the other end of the chimeric molecule, PCR was performed using a bait specific primer that contains a 5’ non-hybridising region representing the complementary Illumina adapter sequence (blue). A two base barcode was incorporated between the 5’ Illumina adapter region and the bait specific primer (yellow box). In a second round of PCR, standard paired-end Illumina PCR primers were used to create tails (green) required to bind to the sequencing flowcell. Using standard Illumina paired-end sequencing primers, the first read determines the prey end of the chimeric molecule. The second read determines the presence of bait in the chimeric molecule, its allelic origin and identifies the sample. The first two bases represent the barcode (CC: B6xSD7 E13.5 liver, GG: SD7xB6 E13.5 liver, AC: B6xSD7 E13.5 placenta, AG: SD7xB6 E13.5 placenta). Following bases correspond to either the B6 or the SD7 bait which are distinct by a SNP at position Chr7:150474522 (NCBIM37, B6: T, SD7: C, yellow star).

Table of primers

| **Primer designation** | **Sequence** | **Description** |
| --- | --- | --- |
| KvDMR-4C-ext | TGGTCTGGCAGGAAGTTAGG | KvDMR1 specific sequence, 5’ biotinylated |
| Csp6I-ia-oe-for | ACACTCTTTCCCTACACGACGCTCTTCCGATCT | End 1 of the Illumina adapter with Csp6I overhang, upper strand, 3’ phosphorothionate linkage |
| Csp6I-ia-oe-rev | TAAGATCGGAAGAGCGTCGTGTAGGGAAAGAGTGT | End 1 of the Illumina adapter with Csp6I overhang, lower strand, 5’ phosphorylated |
| iaGG-KvDMR1-4C oe | CTCGGCATTCCTGCTGAACCGCTCTTCCGATCTGGTGGATTAAGAAACCATTCCTCAG | Other end of the Illumina adapter, barcode, KvDMR1 specific sequence, 3’ phosphorothionate linkage |
| PE PCR Primer 1.01 | AATGATACGGCGACCACCGAGATCTACACTCTTTCCCTACACGACGCTCTTCCGATCT | Standard Illumina paired-end primer (end 1) introducing flowcell compatible overhangs |
| PE PCR Primer 2.01 | CAAGCAGAAGACGGCATACGAGATCGGTCTCGGCATTCCTGCTGAACCGCTCTTCCGATCT | Standard Illumina paired-end primer (end 2) introducing flowcell compatible overhangs |
| PE read 1 sequencing primer | ACACTCTTTCCCTACACGACGCTCTTCCGATCT | Standard Illumina paired-end sequencing primer for read 1 |
| PE read 2 sequencing primer | CGGTCTCGGCATTCCTGCTGAACCGCTCTTCCGATCT | Standard Illumina paired-end sequencing primer for read 2 |

*Linear 4C-Seq method*

The 4C-Seq protocol was adapted from . 2 µg BglII digested 3C material was used for primer extension with Vent (exo-) DNA polymerase (New England Biolabs) and 100 nM biotinylated bait-specific primer in 50 µl reactions. After clean up with AMPure beads (Agencourt), the material was digested with Csp6I and purified with Qiaquick PCR cleanup (Qiagen). Biotinylated products were bound to streptavidin-coated magnetic beads (Dynabeads M-280, Invitrogen) and unbound products removed by the kilobaseBINDER kit (Invitrogen), before additional digestion with Csp6I and PstI to remove the fragment that is directly adjacent to the bait in the linear sequence. Beads were washed with the kilobaseBINDER wash buffer and 10 mM Tris-Cl (pH 8.5). Ligation of adapters containing Illumina paired-end adapter sequence (end 1) was performed with 5 µM adapter in 40 µl reactions using 2000 U T4 DNA ligase (New England Biolabs). Beads were washed in kilobaseBINDER wash buffer, 10 mM Tris-Cl and HotStar PCR buffer (Qiagen). 4C products were amplified using 400 nM each of a bait specific nested primer containing a two base barcode and sequence representing end 2 of the Illumina adapter, and an end 1 adapter specific primer. PCR conditions: 95 °C for 15 min, 35 cycles of 94 °C for 30 s, 60 °C for 30 s, 72 °C for 1 min, then 60 °C for 2 min, 72 °C for 10 min. To introduce flowcell compatible overhangs, PCR was performed using standard Illumina paired-end primers (PE PCR Primer 1.01 and 2.01) with Phusion Polymerase (New England Biolabs). PCR conditions: 98 °C for 30 s, 20 cycles of 98 °C for 10 s, 65 °C for 30 s, 72 °C for 30 s, then 72 °C for 5 min.

*Sequencing and downstream bioinformatic analyses*

4C products were sequenced as paired-end runs on an Illumina Genome Analyzer GAIIx (bait side: 76 bases, prey side 38 bases). Sequence pairs were screened for presence of bait and sorted according to sample (barcode) and allelic origin (SNP in bait region). For each bait (maternal and paternal, both reciprocal crosses and both tissues) around 50 000 sequences were obtained, and the corresponding prey end was mapped using ASAP (see next paragraph). For each barcoded sample three biological replicates were pooled. Data was visualised in SeqMonk (http://www.bioinformatics. babraham.ac.uk/projects/seqmonk) with non-duplicated reads counted per 100 kb window. One inherent problem of 4C sequencing data is that reads can only occur at defined positions in the genome, namely starting at a Csp6I site and running towards a BglII site (theoretically possible read). This makes it impossible to discriminate between genuine duplicates which are due to the presence of multiple 3C fusion products, and duplicates resulting from PCR amplification. To exclude potential PCR bias we have counted duplicates only once, which flattens the proximity profile around the bait to some extent. However, by choosing a large enough window size (100 kb), we retain quantitative information: In regions showing frequent associations with the bait, all or nearly all theoretically possible reads are represented in the sequencing data, whereas in regions showing infrequent associations only a fraction of the theoretically possible reads are found. The sequencing depth of the analysed samples is not sufficient to draw robust quantitative conclusions on *trans* or long-distance *cis* interactions, but it allows direct comparison between short-distance *cis* and *trans*-allelic (homologous) associations, as well as the relative frequencies of non-homologous *trans* versus homologous *trans* associations. To ensure we do not overinterpret the 4C-Seq data, we have limited our conclusions to these analyses.

*Determination of* trans*-allelic 4C reads*

To identify 3C chimeras which harbour a bait fragment from one allele and a prey fragment from the other allele, we developed software to align sequences allele-specifically (ASAP; http://www.bioinformatics.babraham.ac.uk/projects/ASAP). Mouse crosses between B6 and SD7 were used to distinguish between parental alleles. SD7 mice carry *M. spretus* sequence at the distal end of chromosome 7. A ‘partial *M. spretus* genome’ for use in ASAP was assembled using BAC sequences (CH35_ 17P24, CH35_35H19, CH35_76I5, CH35_123K8, CH35_374F13, CH35_391C2). The *M. musculus* (B6) and *M. spretus* sequences differ not only by an array of SNPs, but also by various insertions, deletions, duplications and inversions. ASAP takes all these variations into account and returns a subset of reads which are specific for one particular allele. The prey end sequence reads used as input for ASAP were 38 bases and alignment was performed allowing 2 mismatches. ASAP parameters were: -q --phred64-quals -n 2 -l 38 -k 2 --best --chunkmbs 256. If allele-specific reads were from the opposite allele to the bait, they were classified as *trans*-allelic reads and quantified in SeqMonk (compare Figure 1A). With *trans*-allelic associations being infrequent events, it is even more important to exclude artifacts due to PCR amplification (see above) and to count duplicated reads only once. However, this makes it impossible to give a ratio of *trans* to *cis* associations, as duplicated reads are more likely to be ‘real’ for the more abundant *cis* associations. To remain careful in our data interpretation, we only draw the semi-quantitative conclusion that the vast majority of associations occurs in *cis*.

To compare *trans*-allelic associations between samples, they were quantified by the following method: non-duplicated reads were divided by the number of theoretically possible reads per window to give a percentage of positive (found) 3C fragments. As a complication to rigorous data interpretation, we noticed through analysis of individual SNPs between the *M. spretus* BACs and the SD7 mice that a small subset of *M. spretus* SNPs were not present in SD7. Since a ‘missing SNP’ could wrongly be classified as a *trans*-allelic read, we introduced a confidence ranking for *trans*-allelic reads. Theoretically possible reads harbouring a B6/SD7 SNP which *de facto* occured as both the B6 and the SD7 variant in the sequencing data were classified as highest confidence reads, and the corresponding theoretically possible read was termed ‘stringently informative’. 80 qualifying 3C fragments were identified and used for the comparison of *trans*-allelic associations between samples, and for the comparison of homologous to non-homologous *trans* associations (Figure 1C, D). Since stringently informative reads represent only a subset of all allele-specific reads and are therefore less frequent, we increased the window size to 200 kb for the comparison between samples, and to the complete informative region (1.4 Mb) for the comparison of homologous to non-homologous associations. By using such stringent scoring criteria, we are likely underestimating the number of *trans*-allelic associations, in line with the careful interpretation of our 4C-Seq data.

*References*

1. Cope NF, Fraser P (2009) Chromosome conformation capture. Cold Spring Harb Protoc 2009: pdb prot5137.

2. Schoenfelder S, Sexton T, Chakalova L, Cope NF, Horton A, et al. (2010) Preferential associations between co-regulated genes reveal a transcriptional interactome in erythroid cells. Nat Genet 42: 53-61.

3. Kozarewa I, Ning Z, Quail MA, Sanders MJ, Berriman M, et al. (2009) Amplification-free Illumina sequencing-library preparation facilitates improved mapping and assembly of (G+C)-biased genomes. Nat Methods 6: 291-295.

4. Quail MA, Kozarewa I, Smith F, Scally A, Stephens PJ, et al. (2008) A large genome center's improvements to the Illumina sequencing system. Nat Methods 5: 1005-1010.
